# Targeted deletion of fibrillin-1 in the mouse eye results in ectopia lentis and other ocular phenotypes associated with Marfan syndrome

**DOI:** 10.1242/dmm.037283

**Published:** 2019-01-25

**Authors:** Wendell Jones, Juan Rodriguez, Steven Bassnett

**Affiliations:** 1 Department of Ophthalmology and Visual Sciences, Washington University School of Medicine, 660 S. Euclid Ave, Box 8096, St. Louis, MO 63117, USA; 2 St Louis College of Pharmacy, Department of Basic Sciences, 4588 Parkview Place, St. Louis, MO 63110, USA

**Keywords:** Marfan syndrome, Ciliary zonule, Ectopia lentis, Fibrillin-1, Non-pigmented ciliary epithelium, Lens

## Abstract

Fibrillin is an evolutionarily ancient protein that lends elasticity and resiliency to a variety of tissues. In humans, mutations in fibrillin-1 cause Marfan and related syndromes, conditions in which the eye is often severely affected. To gain insights into the ocular sequelae of Marfan syndrome, we targeted *Fbn1* in mouse lens or non-pigmented ciliary epithelium (NPCE). Conditional knockout of *Fbn1* in NPCE, but not lens, profoundly affected the ciliary zonule, the system of fibrillin-rich fibers that centers the lens in the eye. The tensile strength of the fibrillin-depleted zonule was reduced substantially, due to a shift toward production of smaller caliber fibers. By 3 months, zonular fibers invariably ruptured and mice developed ectopia lentis, a hallmark of Marfan syndrome. At later stages, untethered lenses lost their polarity and developed cataracts, and the length and volume of mutant eyes increased. This model thus captures key aspects of Marfan-related syndromes, providing insights into the role of fibrillin-1 in eye development and disease.

## Abstract (precis)

**Summary:** Targeted knockout of *Fbn1* in the ciliary epithelium of the mouse eye undermines the structural and biomechanical integrity of the ciliary zonule and results in an ectopia lentis phenotype.

## INTRODUCTION

Fibrillin-1 (FBN1) is a large, cysteine-rich glycoprotein of the extracellular matrix. Mutations in *FBN1* underlie Marfan syndrome [MFS; Mendelian Inheritance in Man (MIM): 154700] and the autosomal dominant form of Weill-Marchesani syndrome (AD WMS; MIM: 608328) (Dietz et al., 1991; Comeglio et al., 2002), conditions that severely affect the eye. Ocular manifestations include lens dislocation [ectopia lentis (EL)], microspherophakia, cataract, lens thickening, glaucoma, iris transillumination defects, flattened cornea and axial myopia (Maumenee, 1982; Salchow and Gehle, 2018; Kinori et al., 2017; Konradsen and Zetterström, 2013). EL is almost ubiquitous in WMS patients and is sufficiently common in MFS patients [>60% (Nelson and Maumenee, 1982; Maumenee, 1982)] to be included as one of two cardinal features in the disease nosology (the other being aortic root dilation) (Loeys et al., 2010).

To investigate the roles of *FBN1* mutations in the pathophysiology of MFS and related conditions, the *Fbn1* locus has been targeted extensively in mice. Numerous models have been generated, including hypomorphs, nulls and missense mutations (Sakai et al., 2016). Collectively, these capture key features of human MFS, including kyphoscoliosis, rib overgrowth (Judge et al., 2004) and aneurysms of the ascending aorta (Pereira et al., 1997). None, however, manifests the spectrum of ocular symptoms that characterize MFS and WMS in humans. As a result, we know relatively little about the role of fibrillin-1 in normal eye development or the pathophysiological impact of *FBN1* mutations in MFS or WMS.

EL, the most common ocular symptom of MFS and WMS, results from instability or rupture of the ciliary zonule, a network of fibrillin-rich extracellular fibers that suspends and centers the lens in the eye. The zonular fibers span the narrow gap between the non-pigmented ciliary epithelium (NPCE; located at the inner wall of the eye) and the lens equator. In species that accommodate, the zonule transmits the forces that flatten the lens, bringing distant objects into focus. Each zonular fiber is composed of hundreds or thousands of microfibrils (Raviola, 1971), long filamentous structures ≈10-12 nm in diameter, with a characteristic ‘beads-on-a-string’ appearance when examined by electron microscopy (Kielty, 2017). Partial breakage of zonular fibers manifests as iridodonesis, or tremulousness of the iris (Desai and Tajik, 2017). More profound rupture leads to the complete untethering of the lens (EL).

Proteomic studies indicate that zonular fibers are composed of several dozen proteins, of which fibrillin-1 is by far the most abundant (Cain et al., 2006; De Maria et al., 2017; Eckersley et al., 2018). Other important components include microfibrillar-associated protein-2 (MFAP-2) and latent-transforming growth factor β-binding protein 2 (LTBP-2), each making up about 10% of the proteome by mass. Fibrillin-2, the predominant fibrillin during embryonic development, persists in the adult zonule (Beene et al., 2013). The zonule is also enriched in ADAMTS (a disintegrin and metalloproteinase with thrombospondin motifs)-like proteins (notably ADAMTSL-6 and ADAMTSL-4), which promote microfibril formation *in vitro* (Tsutsui et al., 2010), and cross-linking enzymes such as transglutaminase-2 and lysyl oxidase-like 1, which may stabilize the fibrillar structure.

In the current study, we identified the cells responsible for synthesizing zonular proteins. We then used Cre*-Lox* technology to conditionally disrupt the *Fbn1* locus in selected tissues and examine how fibrillin-1 depletion affected the structural and mechanical properties of the zonular fibers. Critically, conditional *Fbn1* knockout mice developed EL and other sequelae of MFS in humans. As such, the mice constitute a powerful model for testing clinical strategies to treat MFS in the eye.

## RESULTS

### Zonular proteins are produced by NPCE and lens cells

**Fig. 1.**
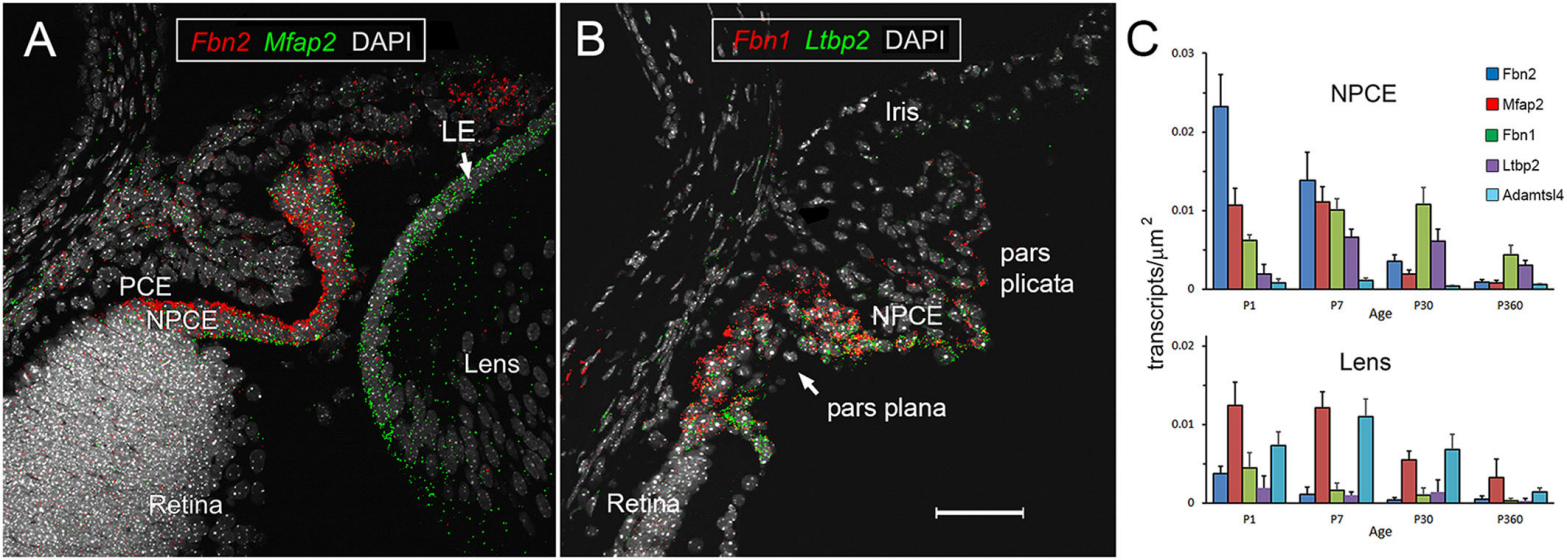
**Expression of genes encoding zonular proteins in ocular tissues during development and aging.** (A) An example of multiplexed fluorescence *in situ* hybridization in the eye of a 1-day-old (P1) wild-type mouse. *Fbn2* transcripts (red puncta) are restricted largely to the non-pigmented ciliary epithelium (NPCE). In contrast, *Mfap2* (green puncta) is expressed in the NPCE and the lens epithelium (LE) at comparable levels. (B) Representative image from a P30 eye probed for *Fbn1* and *Ltbp2* expression. Note that, for both transcripts, the signal is stronger in the pars plana region of the ciliary epithelium than in the pars plicata region. (C) Expression of zonule genes shows marked spatial and temporal fluctuations. *Fbn2*, *Fbn1* and *Ltbp2* are expressed predominantly in the NPCE, *Mfap2* has approximately equal expression levels in NPCE and lens, and *Adamtsl4* is expressed predominantly in the LE. Non-specific binding, assessed using a probe set to a bacterial gene *DapB*, was negligible (data not shown). Data represent mean±s.d. NPCE, non-pigmented ciliary epithelium; PCE, pigmented ciliary epithelium; LE, lens epithelium. Scale bar: 50 µm.

The processes leading to synthesis and assembly of the ciliary zonule are unclear. One possibility is that ocular tissues collaborate in its production. Alternatively, a single tissue could generate the fifty-or-so proteins that constitute the core ‘zonulome’ (De Maria et al., 2017). We used multiplexed fluorescence *in situ* hybridization to monitor the expression of transcripts encoding major zonule components (*Fbn1*, *Fbn2*, *Mfap2*, *Ltbp2* and *Adamtsl4*) (De Maria et al., 2017) in tissues of the anterior mouse eye (Fig. 1). Of the genes examined here, *Fbn2* was expressed most strongly in the eyes of young mice. At postnatal day 1 (P1), *Fbn2* transcripts were abundant and restricted largely to the NPCE (Fig. 1A), consistent with previous reports (Shi et al., 2013a). *Mfap2*, by contrast, was expressed at comparable levels in the NPCE and lens epithelium (Fig. 1A). At later stages, expression of zonular transcripts in the NPCE became concentrated in the pars plana region, the narrow band of cells interposed between the folded pars plicata region and the retina (Fig. 1B). Both *Fbn1*, encoding the most abundant zonular protein in the adult, and *Ltbp2* were expressed strongly in this region of the NPCE at P30. We quantified expression of the various transcripts in the lens and NPCE from P1 to 1 year of age (Fig. 1C). This analysis revealed that *Fbn1*, *Fbn2* and *Ltbp2* were expressed predominantly in the NPCE throughout development, with comparatively low expression levels in the adjacent lens epithelium. *Adamtsl4* was the only transcript to show preferred expression in the lens at all time points. Collectively, these data indicated that, while some zonular proteins (fibrillin-1, fibrillin-2, LTBP-2) were derived largely from the NPCE, others (ADAMTSL-4) were produced solely by the lens, and some (MFAP-2) were synthesized by both lens and NCPE.

### Conditional deletion of *Fbn1* in NPCE (but not lens) cells depletes the zonule of fibrillin-1

Mice homozygous for a *Fbn1* germline deletion die shortly after birth (Carta et al., 2006), precluding their use in studies of zonule synthesis or stability. Therefore, we used the Cre-*Lox* conditional knockout strategy to target the *Fbn1* locus in lens or NPCE, the two tissues flanking the zonule. We first obtained transgenic mice that expressed *Cre* in NPCE or lens. In *Pax6αCre* transgenic mice, *Cre* is expressed in the nasal/temporal segments of the NPCE/retina (Marquardt et al., 2001). Likewise, the *MLR10Cre* transgene directs *Cre* expression to the mouse lens (Zhao et al., 2004). We verified the published tissue-specific *Cre* expression patterns using the mTmG reporter line (Fig. S1) and then crossed the mice with animals carrying a floxed *Fbn1* allele (Cook et al., 2012). Animals derived from the crosses were indistinguishable in appearance and body weight from controls (Fig. S2), suggesting that expression of the *Cre* transgenes and/or the presence of the floxed *Fbn1* allele did not have obvious untoward effects.

The zonule was not noticeably affected in *Fbn1^Lox/Lox^;MLR10Cre^Tg/−^;mTmG^Tg/−^* (hereafter referred to as Fbn1-lens) mice (Fig. S3). Radially oriented zonular fibers projected from the folded surface of the NPCE, attaching to the lens at its equator. Fibrillin-1 immunofluorescence was uniform along the length of zonular fibers and in fibers located at different points around the circumference. The gap between the tips of the ciliary processes and the lens equator (approximately 200 µm) was consistent at all radial locations and did not differ from that in age-matched controls.

**Fig. 2.**
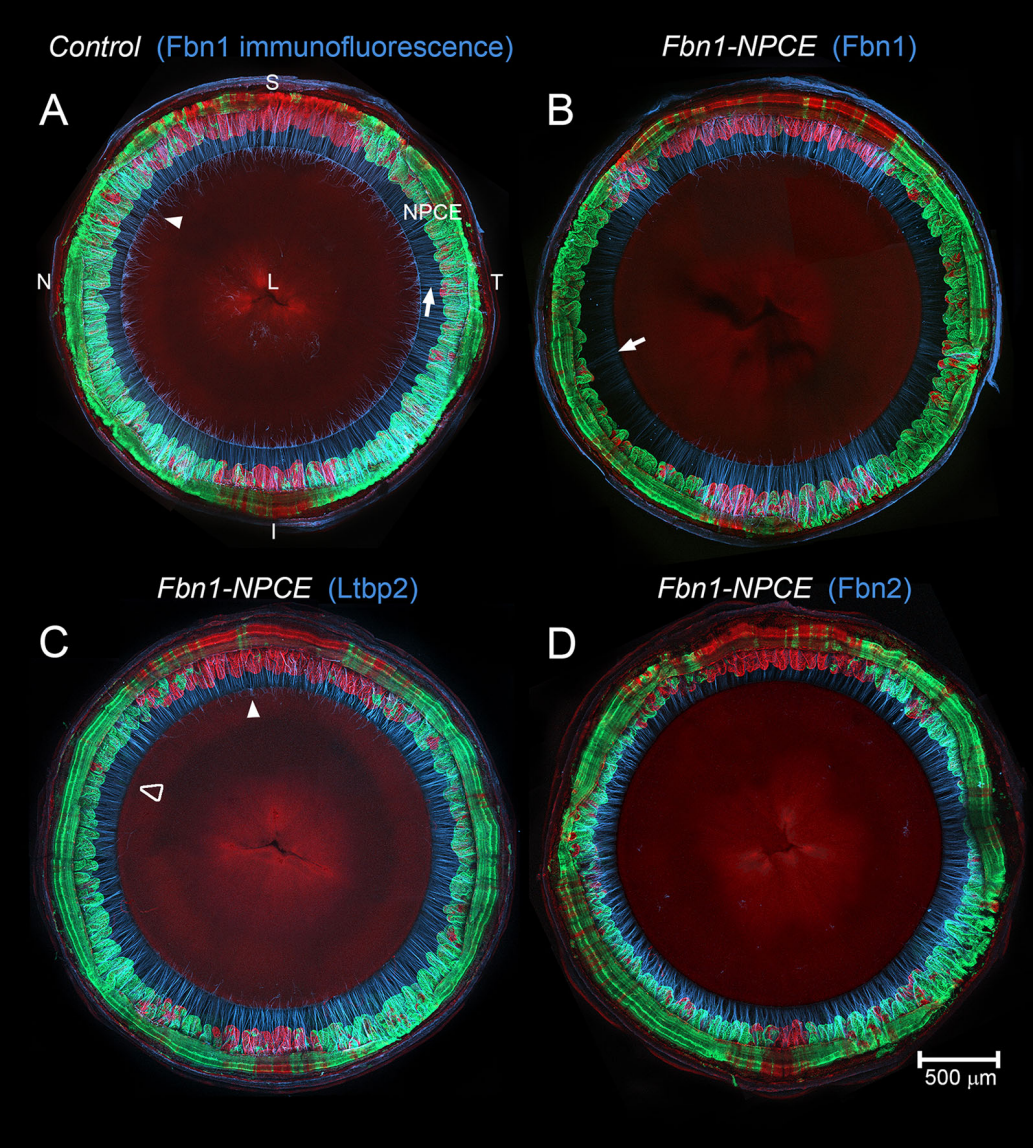
**Conditional deletion of *Fbn1* in the nasal/temporal NPCE causes a reduction in fibrillin-1 immunofluorescence in the adjacent zonule and a corresponding increase in fibrillin-2 immunofluorescence.** In this and other images, the interior of the eye is viewed from the posterior aspect (the retina and posterior sclera having been removed). (A) In 1-month-old control mice, the large, centrally located lens (L) is suspended from the non-pigmented ciliary epithelium (NPCE) by the ciliary zonule (light blue and arrow). *Cre* expression (green) is restricted to the nasal (N) and temporal (T) segments of the NPCE. *Cre*-negative regions (red) are located superiorly (S) and inferiorly (I). Fibrillin-1 immunofluorescence is equally strong in zonular fibers projecting from the *Cre*-positive and *Cre*-negative regions of the NPCE. Fibers attach at the lens equator with some elements (posterior extensions) running a short distance along the posterior lens surface before terminating (arrowhead). (B) In Fbn1-NPCE mice, fibrillin-1 immunofluorescence is markedly reduced in fibers projecting from the *Cre*-positive nasal/temporal segments of the NPCE, although residual immunofluorescence is present near the lens surface (arrow). Note that fibrillin-1 immunofluorescence persists in fibers projecting from *Cre*-negative regions located superiorly and inferiorly. (C) Despite the nominal absence of fibrillin-1, fibers projecting from the nasal and temporal regions have a relatively normal appearance, as judged by immunofluorescence labeling using an antibody to LTBP-2, another abundant zonular protein. Note that, in the *Cre*-positive regions, the posterior extensions of zonular fibers on the lens surface are eliminated (open arrowhead) but persist in *Cre*-negative regions (filled arrowhead). (D) Fibrillin-2 immunofluorescence exhibits a reciprocal pattern to fibrillin-1 (compare D with B), with relatively strong labeling in *Cre*-positive regions and weak labeling in *Cre*-negative regions. Note the absence of fibrillin-2-positive fibers on the surface of the lens, suggesting an uneven distribution of fibrillin-2 proteins along the length of fibers. *N*≥3 eyes per genotype and condition. All staining patterns were consistent between eyes and animals of the same genotype.

In *Fbn1^+/+^;Pax6αCre^Tg/−^;mTmG^Tg/−^* mice (hereafter called controls), all fibers were labeled strongly with antibodies against fibrillin-1 (Fig. 2A). In contrast, in *Fbn1^Lox/Lox^;Pax6αCre^Tg/−^;mTmG^Tg/−^* mice (hereafter referred to as Fbn1-NPCE mice), fibrillin-1 immunofluorescence was largely abolished in fibers extending from the *Cre*-positive nasal/temporal segments of NPCE (Fig. 2B) but was preserved in fibers projecting from the *Cre*-negative superior/inferior segments.

Immunolabeling with antibodies against LTBP-2 revealed that, despite the nominal absence of fibrillin-1, zonular fibers were present in the *Cre*-positive nasal/temporal quadrants and at densities approaching those of the superior/inferior quadrants (Fig. 2C). It has been shown previously that upregulation of one fibrillin isoform in the eye may compensate for loss of another (Shi et al., 2013b; Beene et al., 2013). To test whether fibrillin-2 expression was upregulated in the absence of fibrillin-1, eyes of Fbn1-NPCE mice were stained with anti-fibrillin-2. The resulting image showed that fibrillin-2 immunofluorescence was increased in fibers projecting from the *Cre*-positive nasal/temporal regions of the NPCE (Fig. 2D). Based on this observation, we hypothesized that upregulated *Fbn2* expression may compensate for the absence of fibrillin-1.

We used quantitative multiplexed *in situ* hybridization to assess the recombination efficiency in *Cre*-positive regions of the NPCE and determine whether the observed increase in fibrillin-2 immunofluorescence in the nasal/temporal quadrants (Fig. 2D) signified an increase in *Fbn2* transcription in those regions. Compared to age-matched wild types, controls, or *Cre*-negative regions of Fbn1-NPCE mice, *Fbn1* expression levels in *Cre*-positive regions were reduced significantly (by >80%; Fig. S4). In contrast, there was no significant difference in *Fbn2* expression between *Cre*-positive and *Cre*-negative regions of the NPCE in Fbn1-NPCE mice (Fig. S4). Together, these data indicate that *Fbn1* expression was reduced substantially in *Cre*-positive regions and that increased fibrillin-2 fluorescence in the fibrillin-1-depleted zonule was not a consequence of increased *Fbn2* transcription. We note that, in other systems, fibrillin-1 depletion can result in enhanced fibrillin-2 immunofluorescence via antigen unmasking (Charbonneau et al., 2010) and that such a phenomenon could account for the staining pattern observed in Fig. 2D.

Close examination of fibers projecting from *Cre*-positive regions of the NPCE revealed that they were not entirely devoid of fibrillin-1 (Fig. 2B and Fig. S5). At higher magnification, faint fibrillin-1 immunofluorescence was visible in those segments of the zonular fibers proximal to the lens surface. Due to mild mosaicism in transgene expression, small islands of *Cre*-negative cells were sometimes present in the nasal/temporal quadrants (see also Fig. S1). In contrast to the surrounding fibers, zonular fibers projecting from the *Cre*-negative islands were labeled throughout their lengths by the anti-fibrillin-1 antibody (Fig. S5B).

### EL develops in Fbn1-NPCE mice but not in Fbn1-lens mice or controls

**Fig. 3.**
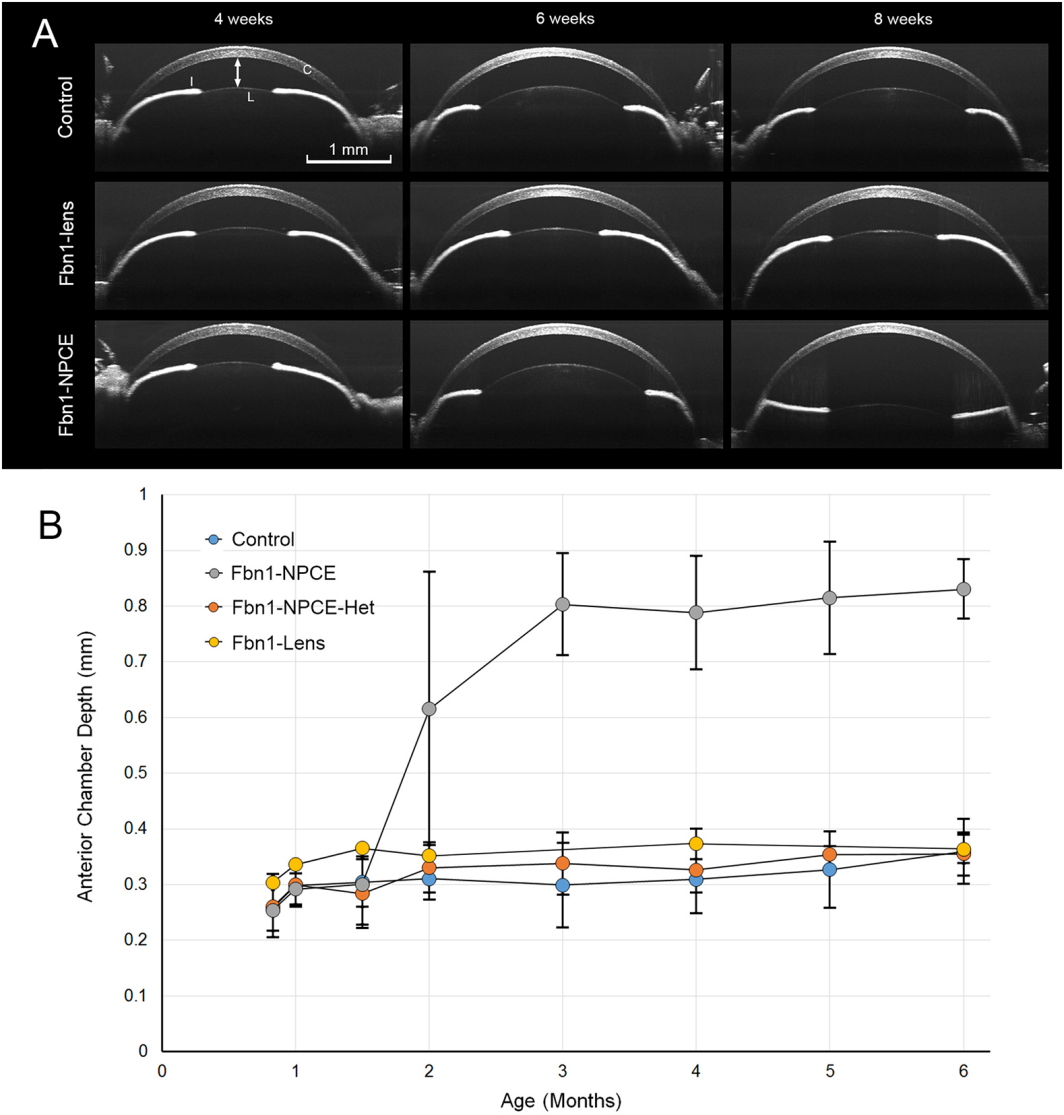
**Onset of EL in Fbn1-NPCE mice.** (A) Anterior segment is visualized by optical coherence tomography (OCT) in living mice. The cornea (C), iris (I) and lens (L) are visible. The distance between the inner surface of the cornea and the anterior surface of the lens is the anterior chamber depth (ACD; double-headed arrow). The position of the lens is stable in control mice or Fbn1-lens mice. In Fbn1-NPCE mice, the lens is initially in the correct position. However, between 6 and 8 weeks of age the lens dislocates backward in the eye (lower right panel). As a result, the ACD increases sharply and the iris, which is normally convex because of the support of the underlying lens, flattens. (B) In Fbn1-NPCE mice, there is a sharp increase in ACD beginning at 6 weeks of age. This reflects the onset of EL, a condition that becomes 100% penetrant by 3 months. Note that EL did not develop in control, heterozygous Fbn1-NPCE mice or conditional lens knockouts. *N*≥10 eyes at all ages. Data represent mean±s.d.

The position of the lens in Fbn1-NPCE, Fbn1-lens or control mice was assessed non-invasively by optical coherence tomography (OCT; Fig. 3). In control animals, the iris had a convex configuration, due to the support of the underlying lens. The central region of the anterior lens surface was faintly visible through the pupil, allowing the anterior chamber depth (ACD; the distance between the inner surface of the cornea and the front surface of the lens) to be measured. Until 6 weeks of age, lens position was indistinguishable between the various genotypes (Fig. 3A). However, between 6 and 8 weeks of age, 8 out of 12 Fbn1-NPCE mice developed unilateral or bilateral EL. By 3 months, all of the mice had developed bilateral EL. Mice were followed for periods of >1 year. Over this period, EL did not develop in control, Fbn1-NPCE-Het (mice heterozygous for the floxed allele) or Fbn1-lens animals. The change in ACD was quantified for the various genotypes (Fig. 3B). Mean ACD increased sharply (from ≈0.3 to ≈0.8 mm) between 6 and 12 weeks of age in Fbn1-NPCE mice only. The imaging depth of the OCT system was 1.6 mm. As a result, the final disposition of ectopic lenses within the eye could not be verified. We overcame this limitation by examining fixed eyes in three dimensions by X-ray microscopy (Fig. S6). The X-ray images of eyes from 3-month-old control mice revealed the relative positions of the lens, ciliary body and iris (Fig. S6A). In age-matched Fbn1-NPCE mice, ectopic lenses were located immediately adjacent to the retina (Fig. S6B). In the absence of the supporting lens, the iris adopted a flattened configuration and the ciliary processes, which are normally angled forward in the eye, were instead oriented perpendicular to the eye wall.

**Fig. 4.**
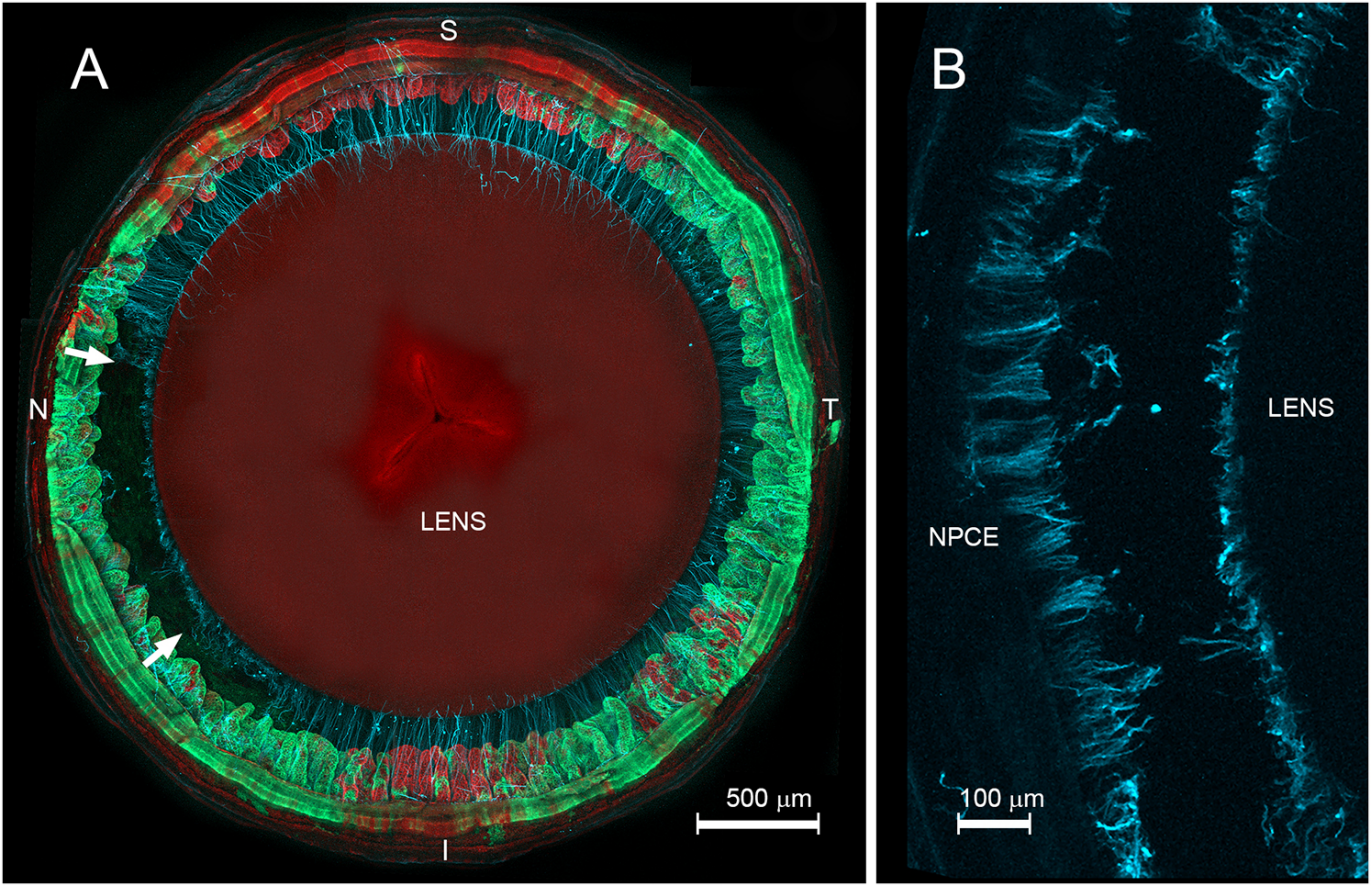
**Breakage of zonular fibers in *Cre*-positive (green) regions precedes lens dislocation.** (A) Eye from an 8-week-old Fbn1-NPCE mouse. The zonular fibers (light blue) are visualized using MFAP-2 immunofluorescence. Note the breakage of a stretch of zonular fibers (between the arrows) projecting from the nasal (N) region of the NPCE. (B) The fibers break near their midpoints, with fiber remnants visible on the surface of the lens and NPCE. N, nasal; T, temporal; S, superior; I, inferior.

ACD measurements suggested that EL began to develop between 6 and 8 weeks of age. Focusing on this period, we processed Fbn1-NPCE eyes for confocal microscopy to determine the pattern of breakage of the zonular fibers. In most cases, we found zonular fibers to be either completely broken or entirely intact. However, in two eyes (out of 12 examined) we identified eyes in which a subset of zonular fibers was broken (Fig. 4A). In those cases, the stretch of broken fibers was located in the fibrillin-1-depleted nasal region. The fact that only rarely did we observe the zonule in the process of breaking suggests that the partially ruptured zonule is mechanically unstable and breakage of the remaining fibers occurs quite quickly. Close observation indicated that the zonular fibers break in mid-span (Fig. 4B) rather than, for example, detaching from anchorage points on the lens or surface of the ciliary processes.

### The tensile strength of the *Fbn1*-depleted zonule is reduced but breaking displacement is not

**Fig. 5.**
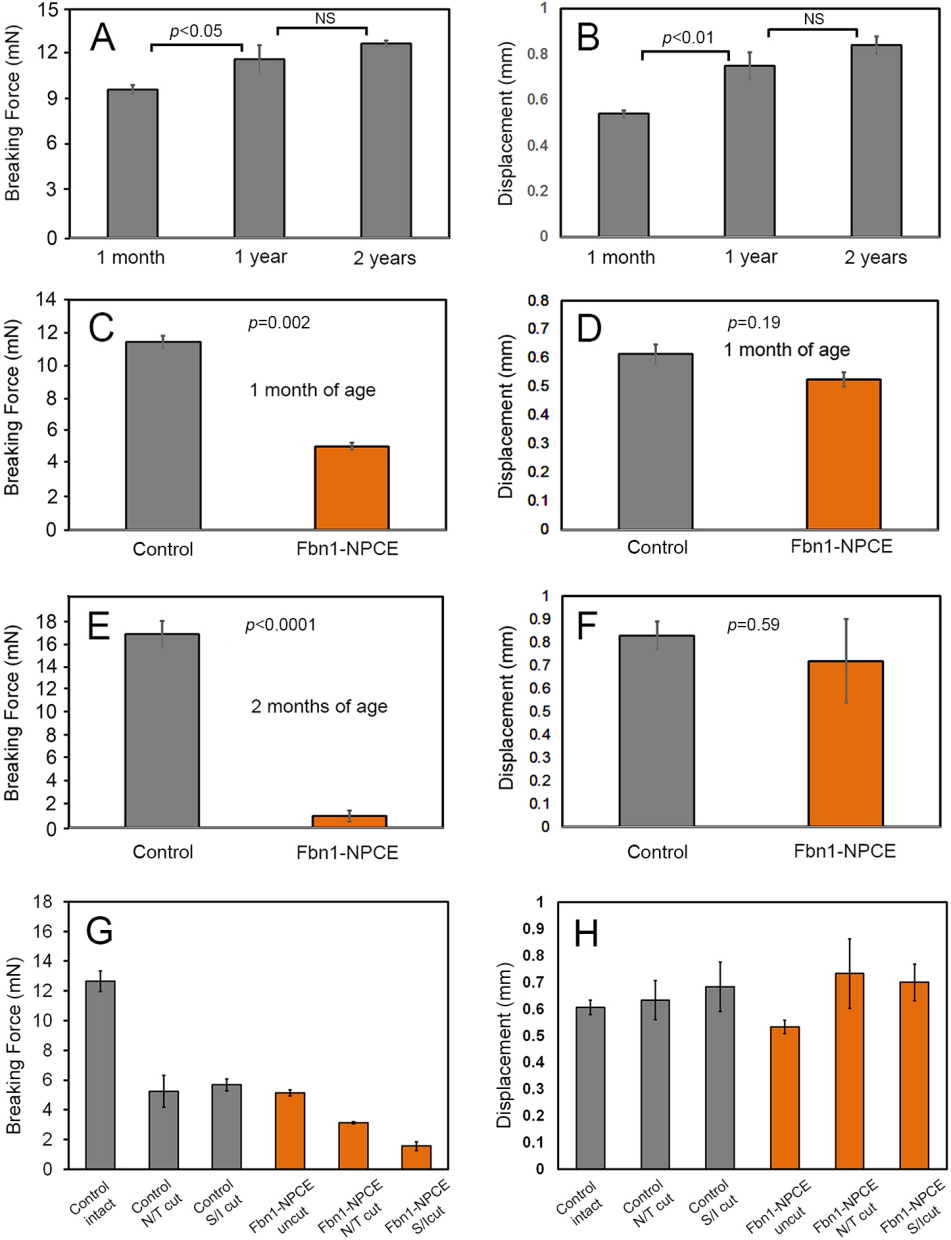
**Biomechanical properties of the ciliary zonule in wild-type, control and Fbn1-NPCE mice.** Tensile strength and breaking displacement were determined using a pull-up assay (see Materials and Methods for details). In wild-type mice, the tensile strength (A) and breaking displacement distance (B) of the zonule increases with age. (C) Compared to 1-month-old control mice, the force required to break the zonule in age-matched Fbn1-NPCE mice is significantly decreased. (D) Displacement distance does not differ significantly between control and Fbn1-NPCE mice. (E) Tensile strength of the Fbn1-NPCE zonule in 2-month-old mice is reduced by >90% compared to controls but the displacement distance is unaffected (F). In Fbn1-NPCE mice, fibrillin-1-rich zonular fibers coexist with fibrillin-depleted fibers. To parse the contribution of fibrillin-1-rich and -deficient fibers to the mechanical properties of the zonule, subsets of fibers were cut in 1-month-old control or Fbn1-NPCE mice. (G) In control mice (gray bars), the tensile strength is reduced by ≈55% if the nasal/temporal (N/T) or superior/inferior (S/I) fibers are cut (*P*<0.01). In Fbn1-NPCE mice (orange bars), the tensile strength of the intact zonule was ≈40% of the control zonule (*P*<0.001). Cutting the S/I fibers in Fbn1-NPCE caused a larger reduction in breakage force than cutting the N/T fibers (*P*<0.01), suggesting that the S/I fibers make a greater contribution to the tensile strength of the mutant zonule. (H) The vertical displacement required to break the zonular fibers was approximately 0.6 mm in all cases. *N*≥3 or more eyes per age and genotype (A-F). *N*≥3 eyes per condition (G and H). Data represent mean±s.d.

To test whether fibrillin-1 depletion affected the tensile strength of the zonule, we used a pull-up technique to measure the force and vertical displacement required to break the zonular fibers of 1-month-old wild-type or mutant mice (Fig. 5). At that age, the appearance of the fibrillin-1-depleted fibers at the level of resolution of the light microscope was indistinguishable from controls (Fig. 2A-C). In wild-type (C57/BL6) mice, fibers broke after approximately ≈10 mN of tensile force were applied (Fig. 5A). Ultimate tensile strength increased substantially (by about 30%) in older (1- or 2-year-old) wild-type animals. The breaking displacement (the vertical distance the lens could be raised from the immobilized eye cup before the zonule ruptured) was also substantially greater in older eyes than younger eyes (0.55 mm and 0.85 mm, respectively; Fig. 5B). In 1-month-old Fbn1-NPCE mice, the breaking force was reduced by ≈55% compared to age-matched controls (Fig. 5C), a significant decrease (*P*=0.002). The reduction in breaking force was not paralleled by changes in displacement distance (Fig. 5D).

In 2-month-old mice, the breaking force for the Fbn1-NPCE zonule was reduced by >90% compared to age-matched controls (Fig. 5E), while the breaking displacement was comparable between genotypes (Fig. 5F). The tensile strength of the zonule in Fbn1-lens mice showed no such reduction (Fig. S7A) and the displacement distance was not significantly different from controls (Fig. S7B). Thus, after disruption of *Fbn1* expression in the NPCE (and not lens), the structural integrity of the zonule was compromised.

In Fbn1-NPCE mice, fibrillin-1-rich zonular fibers coexist with fibrillin-depleted fibers (Fig. 2B,C). The pull-up technique measures the properties of the two types of fibers in parallel. To better distinguish the properties of wild-type (fibrillin-1-rich) and fibrillin-1-depleted fibers, the superior/inferior and nasal/temporal sets of fibers were cut selectively in control or Fbn1-NPCE eyes. In control eyes, cutting the fibers in the nasal/temporal quadrants or superior/inferior quadrants had comparable effects, in both cases reducing the overall breaking force by 50-60% (Fig. 5G). However, in Fbn1-NPCE mice, selectively cutting the superior/inferior fibers caused a larger proportional reduction in tensile strength than cutting the nasal/temporal fibers (70% versus 39%, respectively). We conclude that the residual tensile strength of the mutant zonule is attributable largely to the presence of fibrillin-1-rich zonular fibers in the superior and inferior quadrants. The vertical displacement values were comparable in the two genotypes, whether subpopulations of fibers were severed or not (Fig. 5H).

### Loss of *Fbn1* is associated with reduced fiber density and fewer microfibrils per bundle

**Fig. 6.**
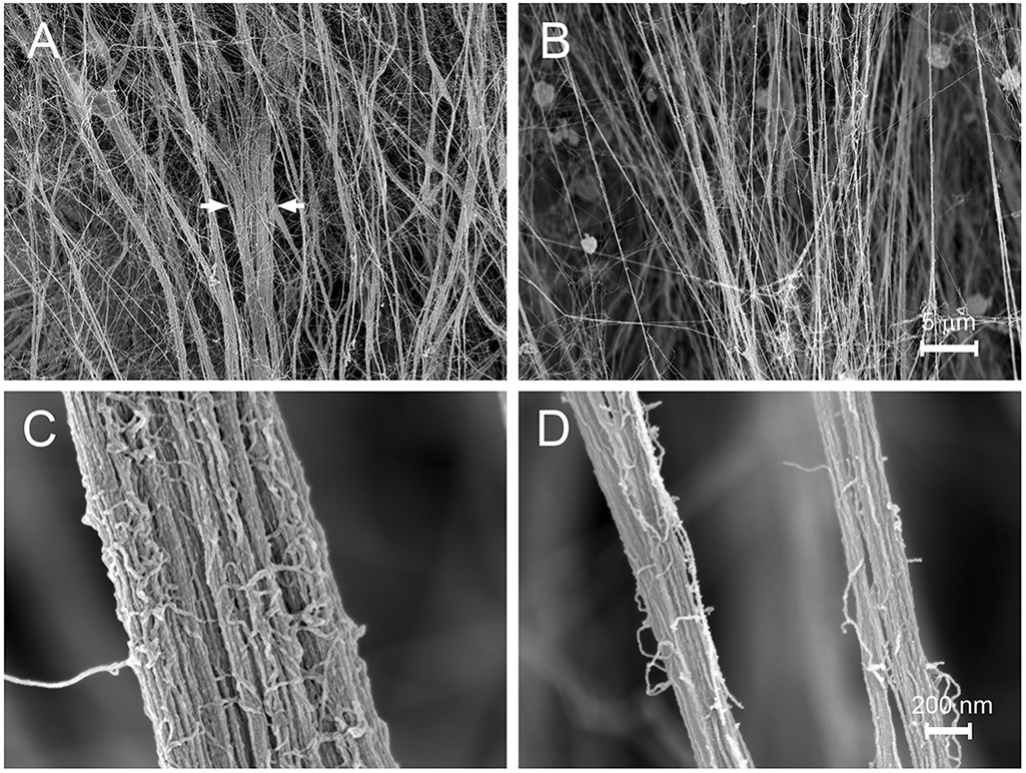
**Ultrastructure of control and Fbn1-depleted zonular fibers, imaged at midspan.** The images are oriented such that the ciliary epithelium is at the top and lens at the bottom (although neither tissue is visible in these high-magnification images). (A,C) Control and (B,D) Fbn1-depleted zonular fibers. (A) As they course toward the lens, control fibers coalesce to form thicker bundles (arrows). (B) In contrast, fibrillin-1-depleted fibers are generally thinner and do not converge to form bundles. (C) At higher magnification, the individual microfibrils that comprise the fibers are visible. Each microfibril is 10-12 nm in diameter. (D) Although thinner on average, zonular fibers in Fbn1-NPCE mice are otherwise indistinguishable from control fibers.

Loss of fibrillin-1 from zonular fibers was associated with a substantial reduction in tensile strength (Fig. 5). To identify structural correlates, we used high-resolution scanning electron microscopy (SEM) to examine the zonule in 1-month-old control mice and the *Cre*-positive and *Cre*-negative regions of Fbn1-NPCE mice (Fig. 6).

The wild-type zonule consisted of a fine web of radially oriented fibers (Fig. 6A). The fibers emerged from a fibrous cladding on the surface of the ciliary processes and spanned the 200-300 μm gap between the NPCE from the lens equator. Converging towards the lens, fibers commonly coalesced into larger bundles. These were notably absent from fibrillin-1-deficient regions of Fbn1-NPCE eyes (Fig. 6B), where, instead, individual fibers projected from the NPCE directly to the lens. Imaged at their midpoints, fibers in control eyes or the superior (*Cre*-negative) regions of Fbn1-NPCE eyes (Fig. 6C) were substantially thicker than fibrillin-1-depleted fibers (Fig. 6D), although a wide range of fiber diameters was present in all cases. Fibrillin-rich and fibrillin-depleted fibers were both composed of microfibrils, visible at high magnification. The diameter of the component microfibrils did not differ significantly between the *Cre*-positive and *Cre*-negative regions of Fbn1-NPCE eyes [diameter=10.8±1.8 nm (mean±s.d.; *n*=33) for Fbn1-depleted fibers, 10.5±2.3 nm (*n*=30) for Fbn1-rich fibers, and 10.65±1.9 nm (*n*=141) for controls].

In addition to affecting fiber diameter, depletion of fibrillin-1 in the NPCE had a marked effect on the appearance of the ciliary processes (Fig. S8). In control mice, the surface of the processes was obscured by the presence of a loose basket weave of orthogonally oriented microfibril bundles (Fig. S8A,B). This microfibril cladding was largely absent from *Cre*-positive regions of the ciliary epithelium in Fbn1-NPCE mice (Fig. S8C,D).

**Fig. 7.**
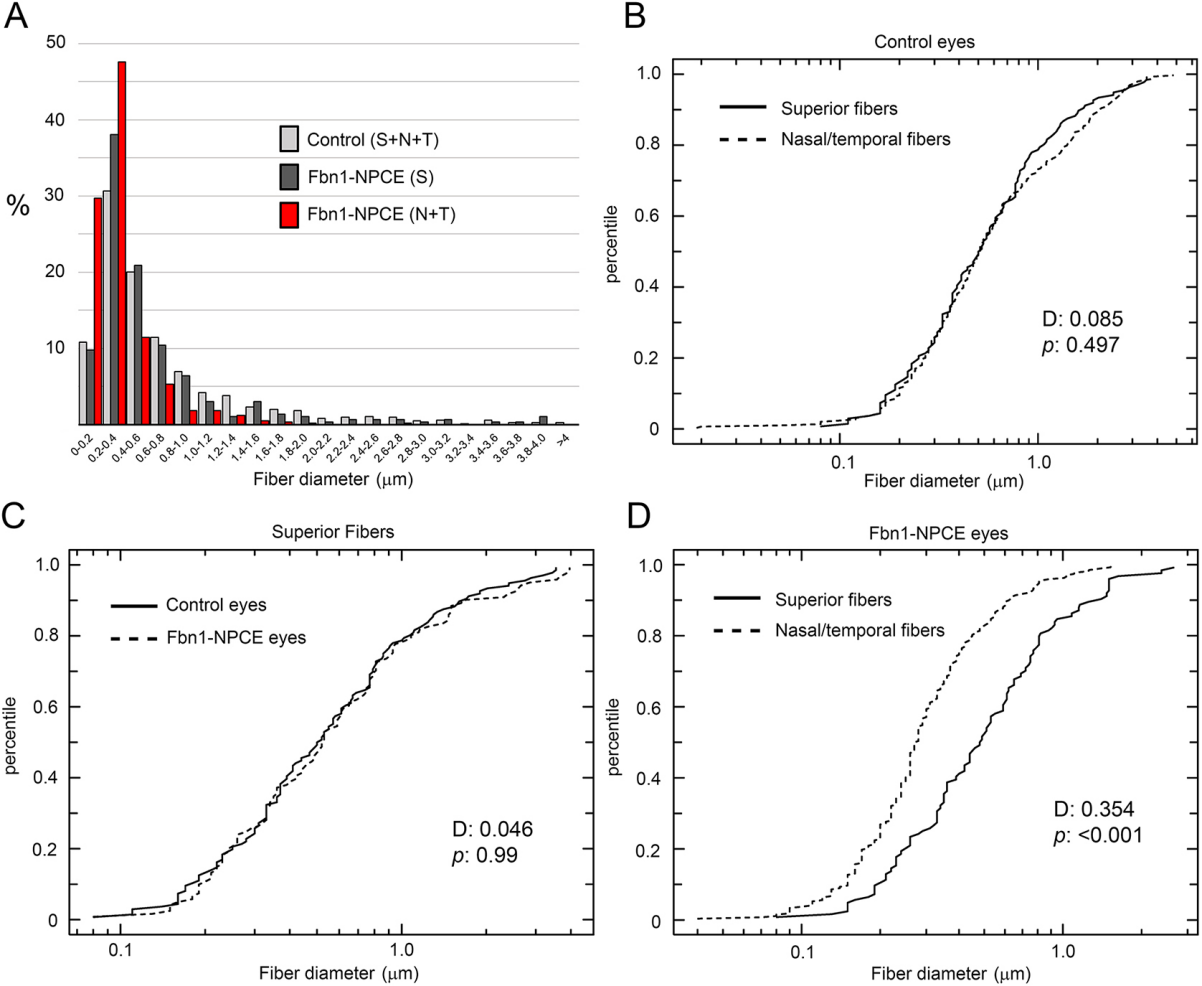
**Zonular fiber diameter is reduced in Fbn1-depleted regions.** Fiber diameter was measured by SEM in randomly selected fibers from the superior and nasal/temporal quadrants of control and Fbn1-NPCE mice. The resulting size distributions were evaluated using the two-sample Kolmogorov–Smirnov test. (A) Histogram of fiber size distributions in the three samples. Note that all distributions are positively skewed and that a higher proportion of small-diameter fibers is observed in the nasal/temporal (*Cre*-positive) region of Fbn1-NPCE eyes. (B) Cumulative frequency plots of fiber diameters in the superior and nasal/temporal regions of control eyes. There is no significant difference in fiber diameters between the two regions. (C) Similarly, there is no significant difference in diameter between superficial fibers in control eyes and Fbn1-NPCE eyes. (D) However, fibers in the nasal/temporal region of Fbn1-NPCE eyes are significantly thinner than fibers in the superior region of the same eyes. S, superior; N, nasal; T, temporal; D, Kolmogorov–Smirnov statistic (i.e. maximum difference between the cumulative distributions).

To quantify the effect of fibrillin depletion on zonular fiber structure, we used SEM to measure the diameter of several hundred randomly selected fibers from control mice, and the *Cre*-positive (nasal/temporal) and *Cre*-negative (superior) regions of Fbn1-NPCE mice (Fig. 7). For each genotype, a broad distribution of fiber diameters was recorded, ranging from 0.1 to 4.0 μm (Fig. 7A). This range of fiber widths corresponds to <100 microfibrils per fiber to >100,000 microfibrils per fiber [under the assumption that fibers are circular in cross-section, microfibrils are 11 nm in diameter and microfibril packing is efficient (i.e. a hexagonal lattice)]. The fiber size distributions were positively skewed in all cases, with a high proportion of small-diameter fibers and relatively few large (i.e. >1.0 µm) fibers. The size distribution was similar in control eyes or fibers emanating from the superior regions of Fbn1-NPCE eyes. In contrast, compared to control eyes or *Cre*-negative regions, the size distribution of fibers emanating from *Cre*-positive nasal/temporal regions was skewed toward smaller-diameter fibers. For example, ≈30% of fibers from the nasal/temporal regions had diameters <0.2 µm, whereas only 10% of control or *Cre*-negative fibers fell in this range. Similarly, large-diameter fibers were comparatively rare in the fibrillin-1-depleted nasal/temporal regions. Thus, fibers with diameters >1 µm accounted for 20% (174/868) and 14% (43/297) of control and *Cre*-negative Fbn1-NPCE fibers, respectively, but only 4% (25/603) of *Cre*-positive NPCE fibers. The size distributions of fibers emanating from various quadrants of control and Fbn1-NPCE eyes were compared using the Kolmogorov–Smirnov test (Fig. 7B-D). There was no significant difference in the diameter of fibers projecting from the superior and nasal/temporal regions of the NPCE in control eyes (Fig. 7B). Similarly, there was no significant difference in fiber diameter between the superior region of control eyes and the superior (i.e. *Cre*-negative region) of Fbn1-NPCE eyes (Fig. 7C). Finally, we compared the diameters of fibers emanating from the superior (*Cre*-negative) region of Fbn1-NPCE eyes with those projecting from the nasal/temporal (*Cre*-positive) region of the same eyes (Fig. 7D). Fibers from the *Cre*-negative region had a mean diameter of 0.61 μm and a median diameter of 0.48 μm (*n*=123). In comparison, fibrillin-1-depleted fibers emanating from the *Cre*-positive region had a mean diameter of 0.35 μm and a median diameter of 0.27 μm (*n*=267). The Kolmogorov–Smirnov statistic, *D*, was 0.35 and the null hypothesis (that fibers from the *Cre*-positive and *Cre*-negative regions share a common probability distribution) was rejected (*P*<0.001). Thus, fibrillin-1 depletion in the nasal/temporal quadrants of Fbn1-NPCE mice was associated with significant thinning of the zonular fibers emanating from those regions. We also noted that fiber density (i.e. the number of fibers per microscopic field) was also markedly reduced (by approximately 50%), although we did not attempt to quantify that effect. In summary, morphometric analysis suggests that the zonular fiber density was reduced in fibrillin-1-depleted areas compared to fibrillin-1-rich areas and that the fibers were significantly thinner.

### Aging Fbn1-NPCE mice develop additional Marfan-like ocular symptoms, including cataract and axial elongation

**Fig. 8.**
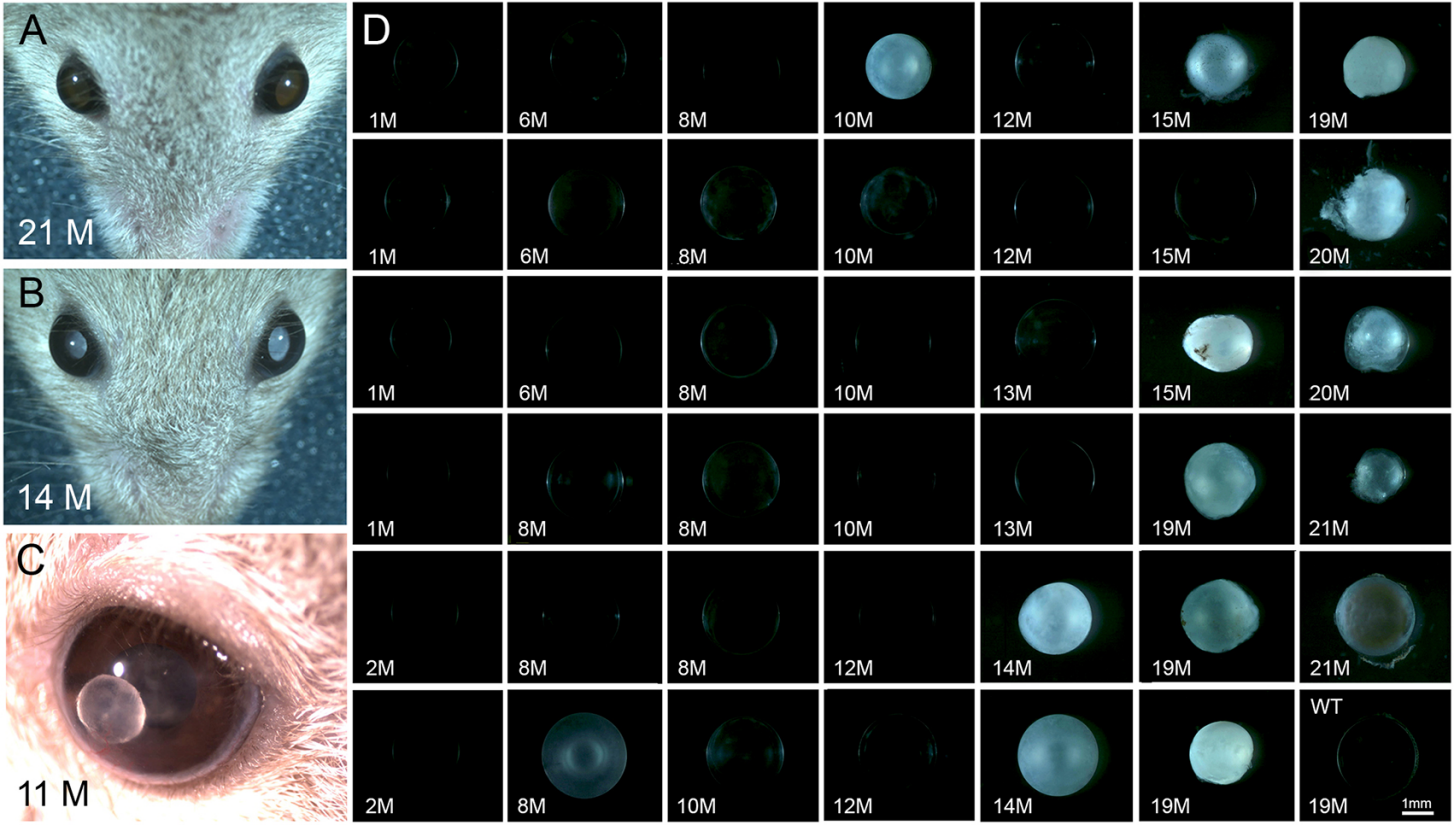
**Ectopic lenses become cataractous.** (A) In control mice, lenses retain their clarity, even in aged animals (see also lower right panel in D). (B) In aged Fbn1-NPCE animals, ectopic lenses often lose their transparency. (C) In some cases, the lens cortex disintegrates and the lens nucleus is expressed through the pupil into the anterior chamber. (D) Gallery showing the effect of age on the prevalence of cataract in Fbn1-NPCE mice. Cataracts are rare in young animals but increasingly common in aged Fbn1-NPCE mice. M, month; WT, wild type.

In control mice, lenses generally remained transparent, even in aged animals (Fig. 8A). EL developed in 100% of Fbn1-NPCE eyes by 3 months of age (Fig. 3B). Initially, dislocated lenses retained their transparency but, as the animals aged, ectopic lenses often became opaque (Fig. 8B-D). Faint cataracts were first noted at 6 months. Thereafter, prevalence increased such that, by 1 year, 86% of eyes from Fbn1-NPCE mice had dense cortical cataracts. In some cases, the cataractous lenses disintegrated, leading to the expulsion of the lens core, which was expressed through the pupil and visible in the anterior chamber (Fig. 8C).

Analysis of the ectopic lenses (Fig. S9B-L) showed a range of histopathological changes, including liquefaction of the cortical layers and, in extreme cases, rupture of the lens capsule. In all cases, the cataractous lenses were smaller than those of age-matched controls (Fig. S9A). An early aspect of the lens phenotype was the overgrowth of the epithelium. The lens epithelium is normally restricted to the anterior surface but, in mutant animals, often expanded to envelop the entire lens (Fig. S10A). Proliferating lens cells, which in wild-type lenses are restricted to the germinative zone (a region of the epithelium immediately anterior to the lens equator), were instead distributed across the anterior and posterior surface of Fbn1-NPCE lenses (Fig. S10A-C). These data suggest that tissue polarity was disturbed in the ectopic lenses.

**Fig. 9.**
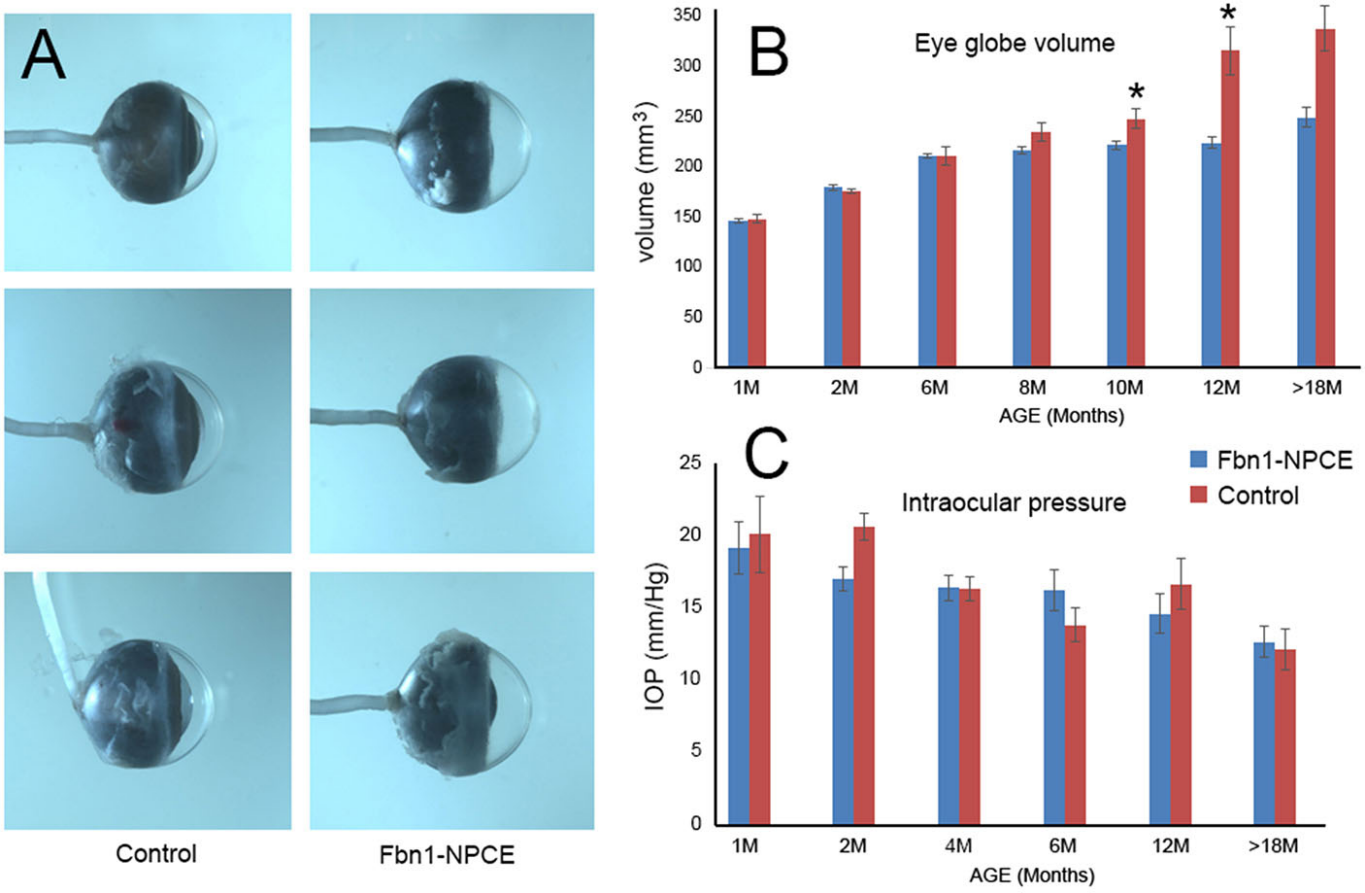
**Eyes from aged Fbn1-NPCE mice have a larger volume than age-matched controls.** (A) Representative examples of eyes from 10-month-old Fbn1-NPCE or control mice showing increased axial length and corneal hazing in the knockout animals. (B) Aggregated data show a difference in ocular volume that is evident at 8 months and statistically significant (*P<0.05) by 10 months (data represent mean±s.d.; *n*≥6 eyes at each time point). (C) For both types of mice, IOP declines with age but differences between genotypes are not significant (*n*≥6 eyes at each time point).

Gross observation suggested that the volume of the eye globe was often increased in Fbn1-NPCE mice compared to controls (Fig. 9A), a finding confirmed subsequently by biometry. Beginning at about 8 months of age, eyes of Fbn1-NPCE mice had a significantly larger volume (Fig. 9B). This was attributable to an increase (of about 14%) in axial length of the globe (rather than an increase in equatorial diameter). At later stages, the cornea often became hazy or vascularized in the buphthalmic eyes. In humans, mutations in *FBN1* can result in glaucoma and pressure-induced buphthalmia (Challa et al., 2006). To test whether increases in ocular volume were the result of elevated intraocular pressure (IOP) within the eye, we measured IOP in Fbn1-NPCE and age-matched controls. In both genotypes, there was a modest decline in IOP from ≈18 mm/Hg in young animals to ≈12 mm/Hg in aged animals, but pressure differences between control and mutant eyes did not reach statistical significance (Fig. 9C). On two occasions, a sharply elevated IOP (>50 mm/Hg) was measured in eyes of aged Fbn1-NPCE mice. In both cases, the cataractous lens had ruptured and particulate material was visible by OCT in the anterior chamber (Fig. S11). This condition may be analogous to phacolytic glaucoma in humans, which is similarly associated with release of lens material from hypermature cataracts. The pressure readings from those two eyes were not included in the aggregated data shown in Fig. 9C.

## DISCUSSION

The mouse eye may serve as a useful model for understanding the ophthalmic sequelae of MFS and related syndromes. Here, we developed two conditional knockouts and used them to gain insights into the role of fibrillin-1 in eye development and disease.

### Zonule synthesis

The source of the proteins that make up the zonule is a long-standing question in eye development. Our *in situ* hybridization data suggest that the major structural proteins (fibrillin-1, fibrillin-2, LTBP-2) are most likely derived from the pars plana portion of the NPCE (although transcripts for MFAP-2, another key zonule protein, were equally abundant in lens). Mutations in ADAMTS genes, namely *ADAMTS10/17* and *ADAMTSL4*, cause either syndromic (MIM: 277600; MIM: 613195) or isolated (MIM: 225100) EL, respectively (Ahram et al., 2009; Morales et al., 2009). Recent evidence shows that these proteins are expressed in the lens and they have been shown to promote microfibril formation *in vitro* (Collin et al., 2015; Beene et al., 2013; Hubmacher et al., 2017). Their precise roles in the synthesis and stability of the zonule have yet to be determined, however. Our experiments on *Adamtsl4* confirmed earlier observations that the gene is expressed strongly by equatorial lens cells with low-level expression in the NPCE (Collin et al., 2015). Thus, although the NPCE is the probable source of the major structural components of the zonule, production of the full proteome likely requires the coordinated synthetic activity of the NPCE and lens. With regard to the most abundant zonular protein, fibrillin-1, we generated mice in which the *Fbn1* locus was disrupted in either lens or NPCE. It was only in the latter case that fibrillin-1 immunofluorescence was depleted in the zonule. Similarly, tensile strength and zonular fiber diameter were affected only when *Fbn1* was targeted in NPCE cells, and EL developed in Fbn1-NPCE mice only. These observations lend further credence to the notion that the NPCE is the major site of fibrillin-1 synthesis. We noted that, in Fbn1-NPCE mice, residual fibrillin-1 immunofluorescence was detected in segments of the zonular fibers proximal to the lens. Fibrillin-1 incorporated at this location may originate in the lens (where *in situ* hybridization analysis identified weak *Fbn1* expression early in development). The generation of double-conditional-knockout (lens plus NPCE) mice would be necessary to test this hypothesis.

The relative expression levels of the various zonule genes fluctuated over time. For example, *Fbn2* was expressed strongly in young eyes, whereas *Ltbp2* transcripts were undetectable at P1 but abundant by 1 month of age. This implies an age-dependence to the molecular composition of zonular fibers. Presumably, fibers synthesized early in life will be enriched in fibrillin-2, whereas those produced later will contain a higher proportion of LTBP-2. The impact (if any) of such compositional fluctuations on zonule biomechanics remains to be determined. The observation that *Fbn1* transcripts were abundant in the NPCE of aged mice allows the possibility that microfibril components turn over with time. Our data are thus consistent with early *in situ* hybridization and radiolabeling experiments, which suggested that zonule synthesis continues, albeit at reduced levels, in the aged eye (Hanssen et al., 2001; Gloor, 1973). Pulse-chase experiments would be required to test this hypothesis directly. Such studies are of particular interest because they speak to whether repair of zonular fibers is possible in MFS patients with compromised zonules.

Although fibrillin-1 is the major structural component of microfibrils, conditional knockout of *Fbn1* in the NPCE did not completely eliminate the zonular fibers. There are several explanations for this somewhat surprising finding. First, as a result of the segmental expression pattern of the *Pax6αCre* transgene, *Fbn1* expression in the superior and inferior regions of the knockout eye was unaffected. Thus, even in knockout eyes, a substantial contingent of wild-type fibers remained. Fibrillin-1 produced in the *Cre*-negative superior and inferior regions of the NPCE may have been incorporated into zonular fibers in the *Cre*-positive nasal and temporal regions. It is also likely that *Fbn1* transcription was not eliminated completely in the *Cre*-positive regions. There was a small degree of mosaicism in *Cre*-transgene expression, and *Cre*-mediated recombination may not have been 100% efficient, as suggested by the *in situ* hybridization experiments. Finally, it is clear for this and earlier studies that fibrillin-2 is expressed in the zonule. Although we did not find evidence of a compensatory increase in *Fbn2* transcription, extant fibrillin-2 (in combination with residual fibrillin-1 expression), may have been sufficient to produce a zonule that was initially serviceable but failed at later time points.

### Structural and mechanical properties

We used a novel pull-up technique (Fig. S12) to measure the ultimate tensile strength of the mouse zonule. The force required to break the Fbn1-NPCE zonule in 1-month-old mice (about 5 mN) was less than half that required to break the zonule of age-matched control eyes. By 2 months of age, the tensile strength of the mutant zonule was only 10% that of controls. Due to the presence of a contingent of wild-type fibers (in the superficial and inferior quadrants), mechanical measurements on the entire zonule likely overestimated the tensile strength of the fibrillin-1-depleted fibers. The displacement values (about 0.6 mm at 1 month of age) did not differ significantly between genotypes, consistent with the notion that there were fewer fibers in the mutant eyes rather than fibers of a qualitatively different type. SEM provided support for this view. Fibrillin-1-rich and fibrillin-1-depleted fibers had a similar morphology but the size distribution of the fibers was shifted significantly toward smaller diameters in Fbn1-NPCE mice. The number of microfibrils per fiber is a function of the cross-sectional area of the fiber, so relatively modest differences in diameter have large effects on the total number of microfibrils present. We also noted decreased fiber density in the Fbn1-NPCE eyes. Together, the shift in fiber diameter and reduced fiber density provides a satisfying explanation for the measured reduction in tensile strength of the mutant zonule.

The displacement distance was approximately 0.6 mm in most cases. The gap between the lens equator and the NPCE is about 0.2 mm. Thus, simple geometry suggests that equatorial fibers stretch by >3-fold before rupturing. Thus, mouse zonular fibers are apparently quite similar in extensibility to human zonular fibers (Assia et al., 1991; Saber et al., 1998). In larger species, where such measurements are practicable, the elastic modulus of the zonule has been estimated. In pig, for example, the modulus is 200-250 kPa (Bocskai et al., 2014), while in humans it is about 300 kPa (Michael et al., 2012). In the mouse, the small size of the eye and the broad distribution of fiber diameters make a direct determination of the elastic modulus problematic. Interestingly, the modulus of the component microfibrils is believed to be orders of magnitude greater than that of the zonule as a whole. Thus, the elastic behavior of zonular fibers probably derives from interactions between microfibrils rather than the elastic properties of the microfibrils themselves.

### Mutational mechanisms

More than 3000 mutations in *FBN1* have been identified in human MFS (http://www.umd.be/FBN1/). Some MFS patients have been identified with unambiguous deletions of an *FBN1* allele (Hilhorst-Hofstee et al., 2011; Mátyás et al., 2007). Significantly, these patients exhibit the full spectrum of symptoms associated with MFS, including EL. Such instances imply that classical MFS can arise via haploinsufficiency (HI). Indeed, recent studies estimate that >30% of adult MFS patients carry HI mutations and there is accumulating evidence that HI mutations may be associated with a more aggressive disease course (Franken et al., 2015). The risk of cardiovascular death, for example, is elevated by 2.5-fold in patients with HI relative to dominant-negative (DN) mutations (Franken et al., 2016, 2017). In Fbn1-NPCE mice, *Cre* expression was directed to the nasal/temporal segments of the NPCE and, as a consequence, fibrillin-1 incorporation into the adjacent ciliary zonule was significantly reduced. Thus, genetically, our mice most closely resemble human MFS patients harboring HI *FBN1* mutations. Mice heterozygous for a germline *Fbn1* knockout do not have an ocular phenotype (Carta et al., 2006). Similarly, in our study, mice heterozygous for the floxed allele did not develop EL. This suggests that, in mice, fibrillin-1 expression in the NPCE must be reduced substantially (i.e. by >50%) to generate an ocular phenotype.

EL was first observed in 8-week-old mice and the EL phenotype was 100% penetrant by 3 months of age. This time course generally parallels that of human MFS patients, where the lens is supported initially by the zonule and EL often manifests during childhood or early adolescence (Maumenee, 1981). In the mice, it is possible that lens dislocation is triggered simply by increased physical activity of the young animals, which would place increasing mechanical demands on the mutant zonular fibers. Alternatively, fibrillin-1-depleted fibers may be more prone to proteolysis during aging. Microfibrils have been shown to serve as substrates for matrix metalloproteases, several of which are expressed in the eye (Sivak and Fini, 2002).

### Ophthalmic sequelae

In human MFS patients, ectopic lenses are normally removed surgically. In the Fbn1-NPCE mice, luxated lenses remained in place, becoming gradually cataractous and in some cases disintegrating completely. Even in transparent dislocated lenses, the fundamental organization of the lens tissue was found to be severely disturbed. The epithelial layer, which is normally restricted to the anterior surface, expanded to envelop the entire lens. In healthy wild-type lenses, S-phase cells are confined to the germinative zone, a swath of the equatorial epithelium (Shi et al., 2014). The germinative zone was unrecognizable in the dislocated lenses, with S-phase cells distributed throughout the epithelium. The eye contains diffusible signals that serve to specify the polarity of the lens (Lovicu and Mcavoy, 2005). One such molecule is fibroblast growth factor (FGF), which is produced by the retina and consequently found at higher concentration in the posterior segment of the eye than the anterior. The FGF gradient triggers fiber cell differentiation at the lens posterior and thus has a critical role in lens polarity. Presumably, morphogen gradients are scrambled for a free-floating lens, leading to dysregulated patterns of lens cell proliferation and differentiation, and, ultimately, opacification.

There was a significant increase in ocular volume at later stages in Fbn1-NPCE mice, attributable largely to an increase in axial length. In emmetropic human eyes, mean axial length is about 23.5 mm (Olsen et al., 2007) compared to 24-25 mm for MFS patients (Drolsum et al., 2015; Kinori et al., 2017; Konradsen and Zetterström, 2013). As a result, MFS patients are often highly myopic. The cause of axial elongation in the Fbn1-NPCE mice is unclear. Clinically, a buphthalmic (enlarged) eye can result from increased IOP, sometimes secondary to lens distintegration (so-called phacolytic glaucoma). We excluded increased IOP as the primary cause of axial elongation in all but two animals. In most cases, IOP was indistinguishable between control and Fbn1-NPCE animals (with or without cataract). Another possibility is that elongation is a response to blurring of the retinal images following the onset of EL. Form-deprivation myopia is a well-known but poorly understood phenomenon in many vertebrate species, including mice (Pardue et al., 2013), and describes a process whereby low-contrast retinal images stimulate scleral growth and axial elongation. The mispositioning of the lens in the Fbn1-NPCE mice would undoubtedly blur the retinal image but it is not known whether this evokes a scleral growth response, especially in adult mice. Finally, it is possible that the enlarged globes observed in aged Fbn1-NPCE mice were the result of edema secondary to an inflammatory reaction triggered by the release of protein from cataractous lenses. Lens-induced uveitis is a well-recognized clinical entity, resulting from an immune response to antigenic material released from ruptured lenses.

In conclusion, we have shown that, in mice, disrupting *Fbn1* expression in the nasal/temporal regions of the NPCE is sufficient to produce EL, cataract and buphthalmia. Because the onset of EL (between 6 and 8 weeks of age) is predictable, and changes in the mechanical properties of the zonule readily quantifiable, these mice represent a useful model for testing clinical interventions aimed at strengthening or preserving the zonular fibers.

## MATERIALS AND METHODS

### Animals

Procedures were approved by the Washington University Animal Studies Committee. Targeted deletion of *Fbn1* in the NPCE and retina was achieved by crossing *Fbn1^Lox^* mice (Cook et al., 2012) with *Pax6αCre* mice (Jackson Lab, Bar Harbor, ME) (Marquardt et al., 2001). In *Pax6αCre* animals, Cre recombinase is expressed from E10.5 in the inner layer of the optic cup (corresponding, in adults, to NPCE and retina) (Marquardt et al., 2001). To visualize the distribution of Cre-expressing cells, *Fbn1^Lox/Lox^;Pax6αCre^+/−^* mice were crossed with the *Cre* reporter strain, *mTmG* (Jackson Laboratory, Bar Harbor, ME) (Muzumdar et al., 2007). On the *mTmG* background, Cre activity causes a shift in expression of fluorescent membrane-targeted proteins, from tandem dimer Tomato (tdTomato) to green fluorescent protein (GFP). Conditional deletion of *Fbn1* in lens was achieved by crossing *Fbn1^Lox/Lox^* mice with *MLR10Cre* mice, which express *Cre* in lens epithelial and fiber cells from E10.5 onward (Zhao et al., 2004). In the text, ‘wild type’ refers to animals on the C57BL/6J background (Jackson Laboratory, Bar Harbor, ME). In all other cases, mutant mice were compared to appropriate ‘controls’ (i.e. littermates of the same genetic background).

### *In situ* hybridization

Mice were killed by CO_2_ inhalation. Tissues were prepared from 1-day-old to 1-year-old mice. Enucleated eyes were fixed for 24 h in 4% RNase-free paraformaldehyde/PBS, embedded in paraffin, and sectioned in the midsagittal plane at 4 µm. Transcripts encoding zonular components were localized by multiplexed fluorescence *in situ* hybridization (RNAscope Multiplex Fluorescence Assay; Advanced Cell Diagnostics, Hayward, CA). The following probe sets were utilized: *Fbn1* (NM_007993.2; bases 2290-3183), *Fbn2* (NM_010181.2; bases 2933-3893), *Ltbp2* (NM_013589.3; bases 5453-6485), *Mfap2* (NM_008546.3; bases 36-1064) and *Adamtsl4* (NM_144899.2; 2624-3725). *In situ* hybridization was also used to visualize *Cre* (KC845567.1; bases 1058-2032) and *GFP* (U55763.1; bases 628-1352) expression in transgenic animals. Each probe consisted of 20 sets of oligonucleotides designed to hybridize to a contiguous ≈1 kb region of target mRNA. Following hybridization and labeling, sections were counterstained with 4′,6-diamidine-2′-phenylindole dihydrochloride (DAPI). As a negative control, adjacent sections were hybridized with *DapB*, a bacterial gene encoding the enzyme dihydrodipicolinate. Pre-mixed *PolR2A* (DNA-directed RNA polymerase II polypeptide A), *Ppib* (peptidylprolyl isomerase B) and *Ubc* (ubiquitin C) probes were used as positive controls. The specificity of target probes was verified using samples from *Fbn1^Lox/Lox^;Pax6αCre^+/−^*, *Ltbp2*^−/−^, *Mfap2*^−/−^ and *Fbn2*^−/−^ mice.

The hybridization/labeling procedure generated a punctate pattern of fluorescence on the tissue sections. The fluorescent puncta corresponded to individual mRNA transcripts (Wang et al., 2012). To gauge the relative abundance of transcripts over time, the number of puncta over the NPCE and equatorial lens epithelium was determined using ImageJ analysis software (Rueden et al., 2017).

### Immunofluorescence

Mice were anesthetized with a mixture of ketamine (80 mg/kg body weight) and xylazine (6 mg/kg), and were perfused transcardially with 4% paraformaldehyde/PBS (pH 7.4). Perfusion fixation preserved the delicate structure of the zonular fibers, while simultaneously stabilizing the wall of the eye. Following fixation, eyes were enucleated and a hole was made in the posterior wall of the globe to facilitate the penetration of fixative. Isolated globes were then immersed in 4% paraformaldehyde/PBS for 24 h. Fixed eyes were washed thoroughly in PBS and the posterior portion of the globe was dissected to the level of the pars plana and discarded. The remaining tissue was incubated in 8% bovine serum albumin (BSA)/PBS blocking solution to minimize non-specific antibody binding. Samples were incubated overnight with primary antibodies in 4% BSA/PBS. After several washes in PBS, samples were incubated with appropriate fluorescently labeled secondary antibodies (1:200) for 2 h at room temperature. Samples were washed in PBS, placed in a glass-bottom dish and imaged by confocal microscopy (Shi et al., 2013a). Nuclei were counterstained with Draq5 (Cell Signaling Technology, Danvers, MA). Eyes from three or more animals were examined. To verify the specificity of antibody labeling, antibodies were tested on eye tissue from germline *Fbn1-*null (Pereira et al., 1997) and *Fbn2-*null (Arteaga-Solis et al., 2001) mice (data not shown).

### Antibodies

Goat-polyclonal anti-MFAP2 was obtained commercially (R&D Systems, Minneapolis, MN) and used at a dilution of 1:100. Anti-mouse fibrillin-1 (pAb 9543; 1:200) and anti-mouse fibrillin-2 (pAb 0868; 1:100) were provided by Dr Lynn Sakai (Oregon Health and Science University, Portland, OR), and anti-LTBP2 was obtained from Tomoyuki Nakamura (Kansai Medical University, Osaka, Japan).

### Ocular phenotyping

#### Gross analysis

Mice were examined under a dissecting microscope for the presence of dislocated lenses, cataracts or buphthalmos (enlarged eyes).

#### Intraocular pressure (IOP)

IOP was measured using a rebound tonometer (iCare TonoLab tonometer, Vantaa, Finland). To avoid injury to the animal or misfiring of the probe, the tonometer was fixed to a stage and anesthetized mice were positioned such that the center of the cornea was aligned with the center of the probe. Three or more measurements were made per eye.

#### Optical coherence tomography (OCT)

The anterior segments of anesthetized mice were visualized non-invasively by OCT (Bioptogen Envisu R2210) using a 10 mm telecentric bore lens with an imaging depth of 1.6 mm and an optical resolution of 2.4 µm. Measurements were made on both eyes and completed within 5 min. Two hundred B-scans (each composed of 1000 A-scans) were averaged to generate the final images. Anterior chamber depth (ACD) was measured along the optical axis from the inner surface of the corneal endothelium to the anterior surface of the lens capsule using software supplied with the instrument.

#### Biometry

The outer dimensions of the globe were determined from digital photos of enucleated eyes. Axial length was measured from the corneal surface to the posterior sclera adjacent to the optic nerve. Equatorial dimensions were measured perpendicular to the axial axis at the widest point. Ocular volume was calculated assuming the globe to be an oblate spheroid.

### Lens optical analysis

To gauge their transparency and refractive properties, lenses were dissected from euthanized mice, placed into pre-warmed tissue culture medium and photographed against a grid pattern. Dark-field illumination was used to assess light scattering within the tissue.

### Confocal microscopy

Images were collected using an LSM510 confocal microscope (Carl Zeiss, Thornwood, NY) or FV1000 confocal microscope (Olympus). Stacks of optical sections were visualized as maximum intensity projections. Using a 10× objective, it was necessary to collect six or more overlapping projections to completely cover the relatively large mouse eye. The projections were then stitched together using Photoshop software to generate the final images.

### Scanning electron microscopy

Eyes from euthanized 1-month-old *Fbn1^+/+^;Pax6αCRE* and *Fbn1^Lox/Lox^;Pax6αCRE* mice were carefully enucleated, fixed for 5 min in 4% paraformaldehyde and dissected down to the level of the pars plana. The dissected eyes were rinsed with PBS to remove any vitreous humor that might adhere to and thereby obscure the zonular fibers. Eyes were then fixed further in 2.5% paraformaldehyde/2.5% glutaraldehyde. Fixed tissue was critical-point dried, sputter coated with gold and imaged using a Merlin–FE scanning electron microscope (Zeiss). The diameters of zonular fibers were determined from the SEM images at 1000× magnification. A small incision in the sclera served as a fiduciary mark, allowing the eye to be oriented in the microscope and the superior, inferior, nasal and temporal quadrants to be identified unambiguously. Differences in the diameters of zonular fibers between wild-type and Fbn1-depeleted regions were assessed using the two-sample Kolmogorov–Smirnov test.

### X-ray microscopy

To visualize the disposition of the lens within the eye, enucleated globes were fixed using a freeze substitution method (Sun et al., 2015) and then incubated in 1:4 dilution of Lugol's iodine solution for 48 h, embedded in 2.0% agarose, and imaged using a Zeiss Xradia Versa 520 microscope. Three eyes from Fbn1-NPCE and aged-matched-control mice were imaged for these experiments.

### Zonule biomechanics

The biomechanical properties of the mouse zonule were measured on fixed (24 h in 4% paraformaldehyde/PBS) tissue (Fig. S12). The corneal surface of the enucleated eye was glued to the base of a custom-fabricated chamber positioned on a sensitive balance (Fig. S12A). The back of the eye was removed by dissection, exposing the posterior surface of the lens, zonule, and the ciliary body. The tip of a small glass probe was brought into contact with the posterior pole of the lens and attached using a drop of Lazer bond™ adhesive. The chamber was then filled with PBS. The probe, which was mounted on a motorized micromanipulator, was raised in 50 µm steps (2 μm/s^2^ acceleration/deceleration) each lasting 10 s. At each step, the associated reduction in sample weight was recorded over a period of 1 min (Fig. S12B). Due to the slow acceleration, we assumed the eye to be in equilibrium and that the measured decrease in weight was equal to the lifting force. In this fashion, the lens was raised steadily from the eye, the zonular fibers becoming increasingly stretched. At a certain point (between position 3 and 4 in Fig. S12B and C), the zonular fibers ruptured and the weight measurements returned to baseline values. Using this apparatus, we measured the tensile strength of the zonular fibers and generated force:displacement curves for each genotype. We also measured the breaking displacement (the distance the lens could be raised from the eye before the zonular fibers failed). Measurements were made on at least three eyes in each case. The person performing the procedure was unaware of the genotype of the mice when making the measurements.

## Supplementary Material

Supplementary information
