# Supplementary material for: Targeted deletion of fibrillin-1 in the mouse eye results in ectopia lentis and other ocular phenotypes associated with Marfan syndrome
Source: Dis Model Mech. 2019 Jan 25;12(1):dmm037283. doi: 10.1242/dmm.037283 (PMC6361150; doi:10.1242/dmm.037283)
Supplement: Supplementary information [file dmm-12-037283-s1.pdf]

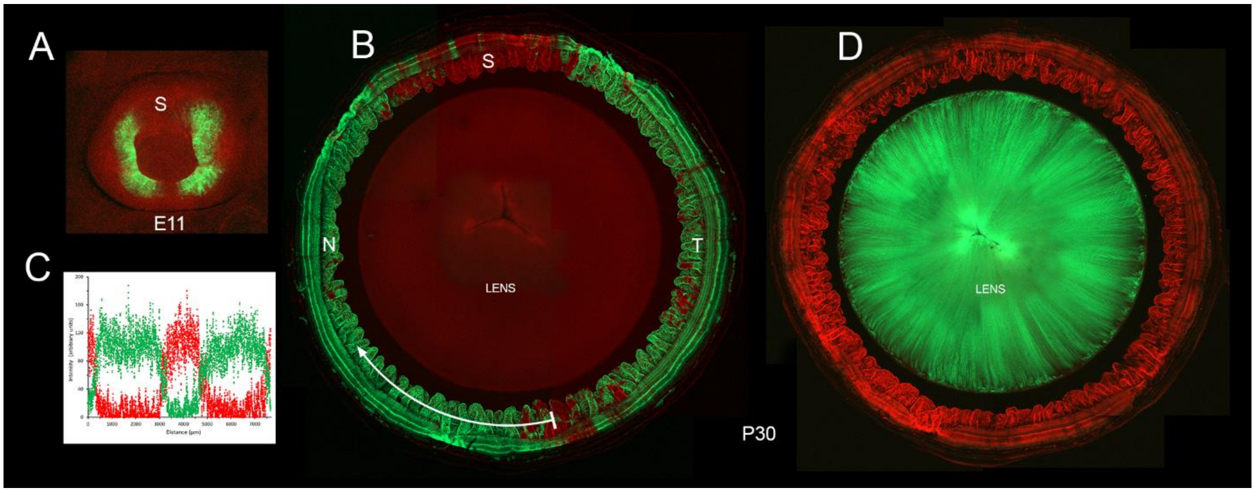

Figure S1. Verifying *Cre* expression patterns in *Pax6αCre* (A-C) and *MLR10Cre* (D) mice. On the mTmG reporter background, *Cre*-mediated recombination causes a shift in expression from membrane-targeted TdTomato (red) to membrane targeted GFP (green). **(A)** The *Pax6αCre* transgene is expressed in the eye from E11 onwards, as confirmed by the presence of GFP fluorescence in the nasal and temporal segments of the inner layer of the optic cup. **(B)** The segmental labeling pattern persists in eyes of 1-month-old mice. Note that the circumferential arc of *Cre*-negative cells in the superior (S) region of the NPCE is larger than the inferior arc. **(C)** A pixel intensity histogram of mTdT (red) and mGFP fluorescence (green) in the NPCE (measured in the direction indicated by the arrow in **(B)**), confirms that the expression pattern is consistent between eyes and that the nasal and temporal *Cre*-positive arcs are longer than the *Cre*-negative superior arc. The inferior arc is the shortest (n=11). **(D)** In *MLR10Cre* transgenic animals, *Cre* activity is restricted to the lens.

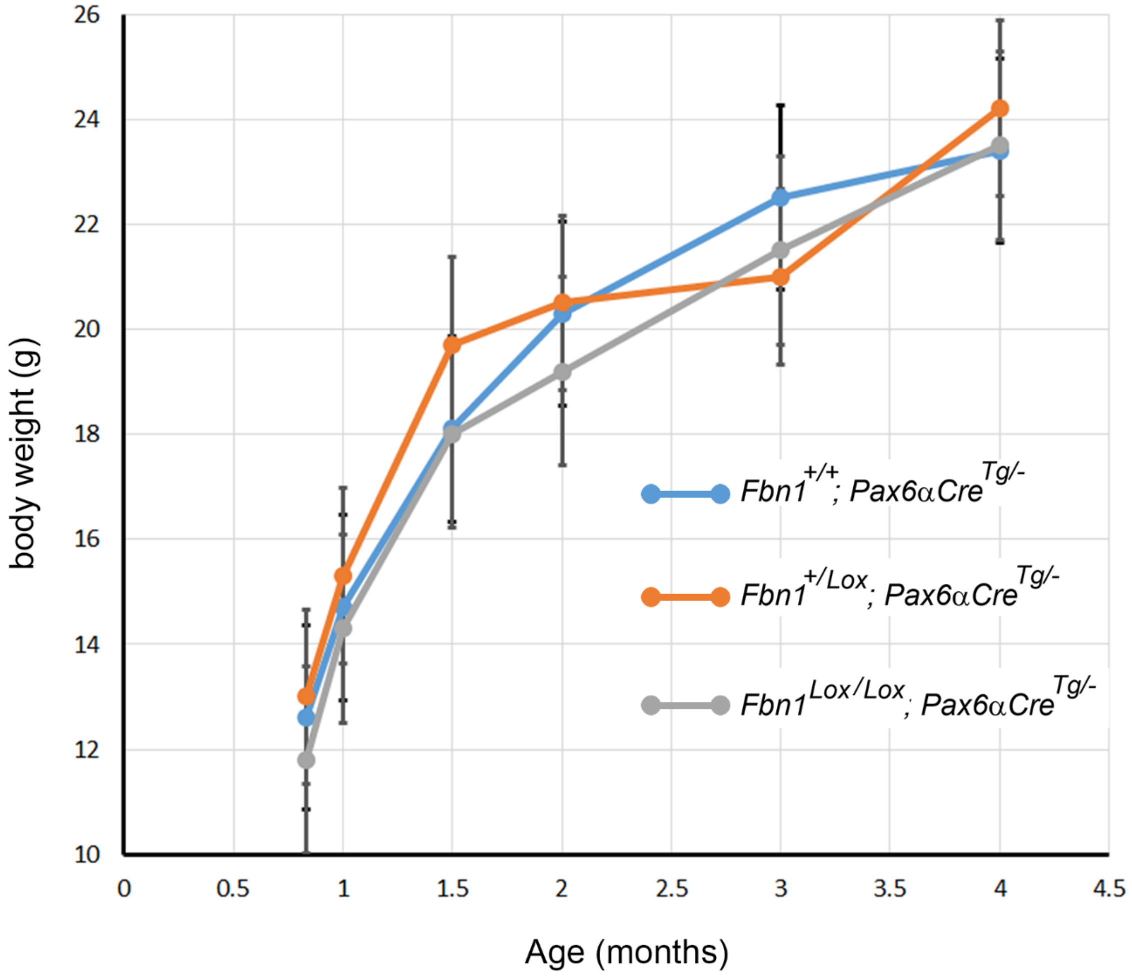

Figure S2. Body weights of control and conditional *Fbn1* knockouts are indistinguishable.

Disease Models & Mechanisms • Supplementary information

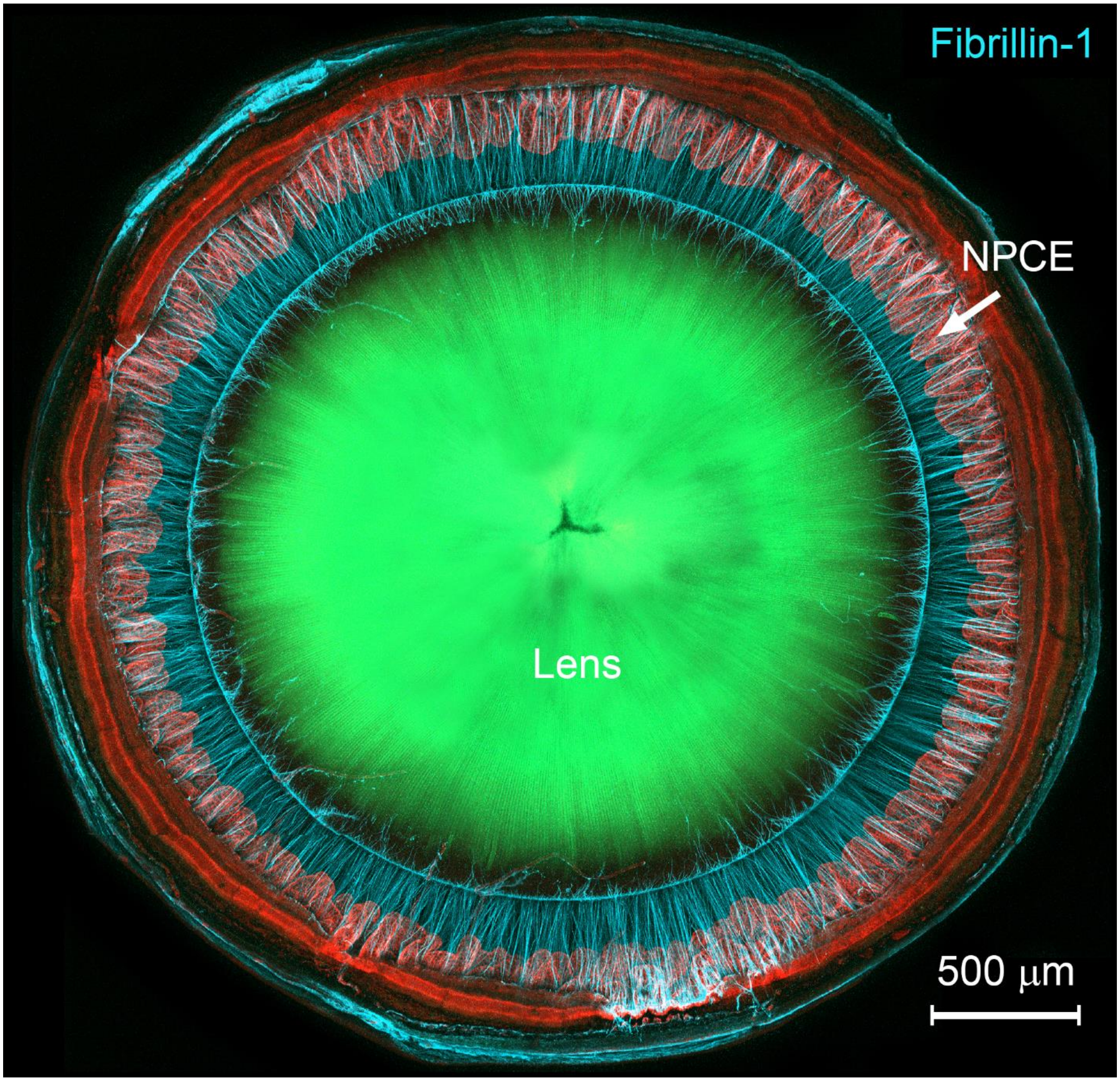

Figure S3. Conditional deletion of *Fbn1* in lens does not prevent fibrillin-1 incorporation into zonular fibers. A network of extracellular fibers (light blue), the ciliary zonule, projects from the folded surface of the non-pigmented ciliary epithelium (NPCE) to the lens equator. *Cre* expression (green) in the lens is marked by a shift from red to green fluorescence (see also Fig. S1). Note that *Cre* is not expressed in the NPCE. Despite conditional deletion of *Fbn1* in the lens, the zonular fibers are intact and strongly immunofluorescent for fibrillin-1. This image is representative of four obtained from Fbn1-lens mice. Scale bar = 500 µm.

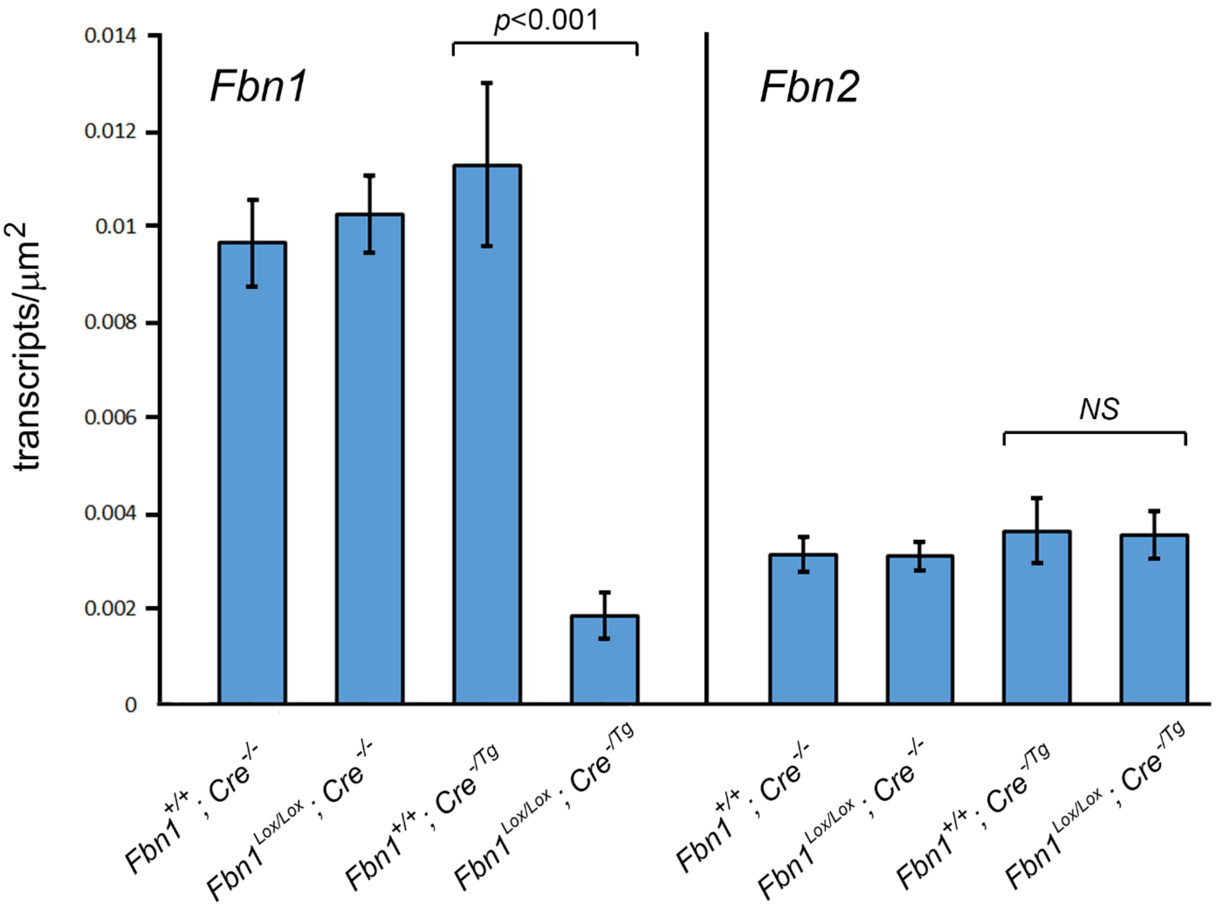

Figure S4. Fibrillin mRNA expression in the NPCE of control or in the *Cre*-positive and *Cre*-negative regions of Fbn1-NPCE mice at 1 month of age. In Fbn1-NPCE mice, *Fbn1* transcript levels are reduced by >80% in the *Cre*-positive region of the NPCE compared to the *Cre*-negative region (p<0.001). No compensatory increase in *Fbn2* expression was detected. In each case, counts were made on at least 3 histological sections from each of three or more mice. Data represent mean ± SD.

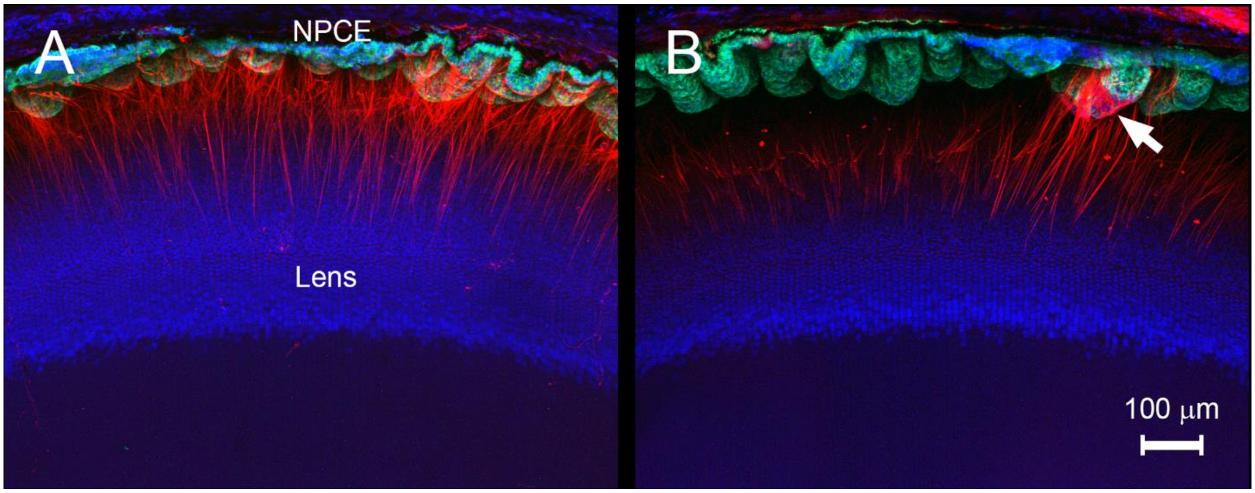

Figure S5. Conditional deletion of *Fbn1* in the NPCE does not eliminate fibrillin-1 immunofluorescence in that portion of the zonular fibers proximal to the lens. (A) In eyes from one-month-old Fbn1-NPCE mice, MFAP-2 immunofluorescence (red) labels the entire length of zonular fibers, from the surface of the nasal/temporal NPCE to the lens equator. **(B)** By contrast, fibrillin-1 immunofluorescence (red) is restricted to that segment of the zonular fibers closest to the lens. An exception is a region of the NPCE containing a small island of *Cre*-negative cells (red, arrow). Fibers emanating from this region are labeled for their entire length. Scale bar = 100 µm.

Disease Models & Mechanisms • Supplementary information

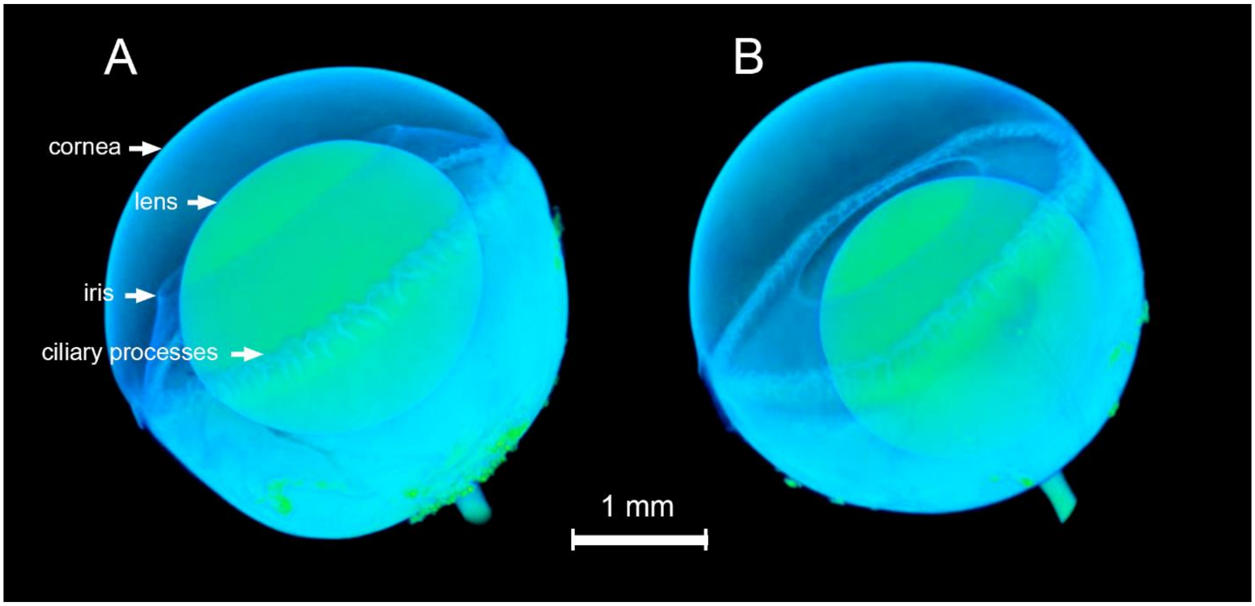

Figure S6. X-ray imaging of ectopic lenses. (A) In eyes from 3-month-old control mice, the central position of the lens (green) is evident. The iris has a convex configuration and the ciliary processes project forward. **(B)** In Fbn1-NPCE mice, EL is accompanied by an increase in anterior chamber depth due to the posterior displacement of the lens (which now is in contact with the retinal surface). In the absence of the supporting lens, the iris and ciliary processes are angled perpendicular to the eye wall. Scale bar = 1 mm.

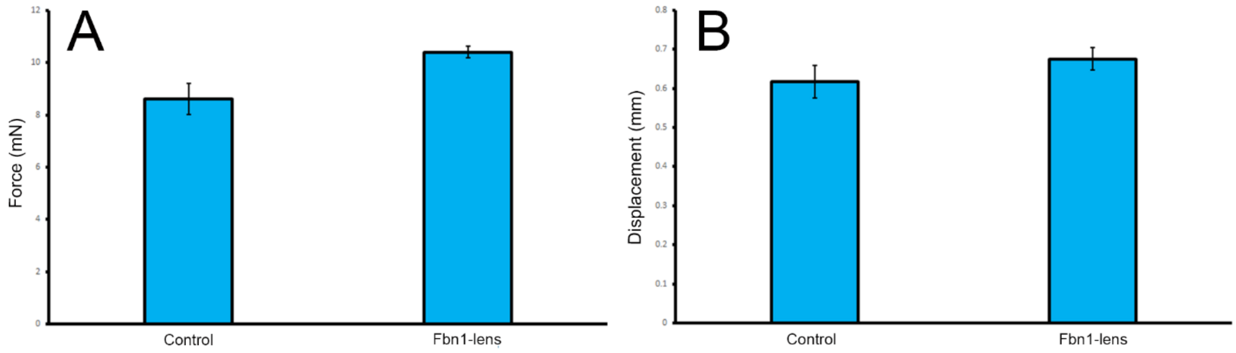

Figure S7. Mechanical properties of the zonule in Fbn1-lens mice. Eyes in which *Fbn1* is conditionally deleted in the lens do not show decreased tensile strength **(A)** or altered displacement distance **(B)** compared to aged-matched (1-month-old) control mice.

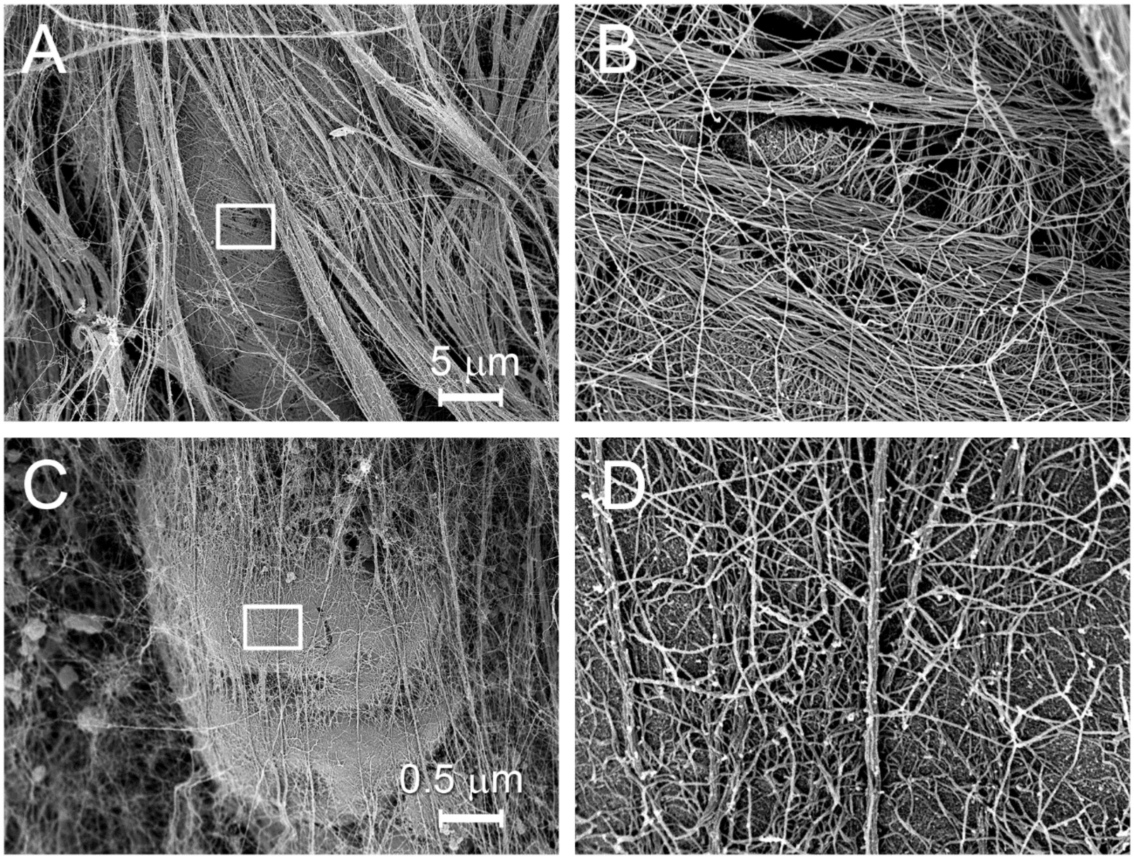

Figure S8. Microfibril cladding covering the surface of the ciliary processes is absent in Fbn1-NPCE mice. (A) In control animals, occasional gaps between zonular fibers allow the surface of the ciliary processes to be visualized (boxed region, shown at high magnification in B). **(B)** A layer of microfibrils, arranged in a loose basket weave pattern, covers the epithelial surface. **(C)** The surface of the ciliary processes in *Cre*-positive regions of Fbn1-NPCE mice is visible, due to the reduced density of zonular fibers. **(D)** At high magnification, there is no evidence of the basket weave cladding found in controls. Scale bar: (**A**) = 5 µm; (**C**) = 0.5 µm.

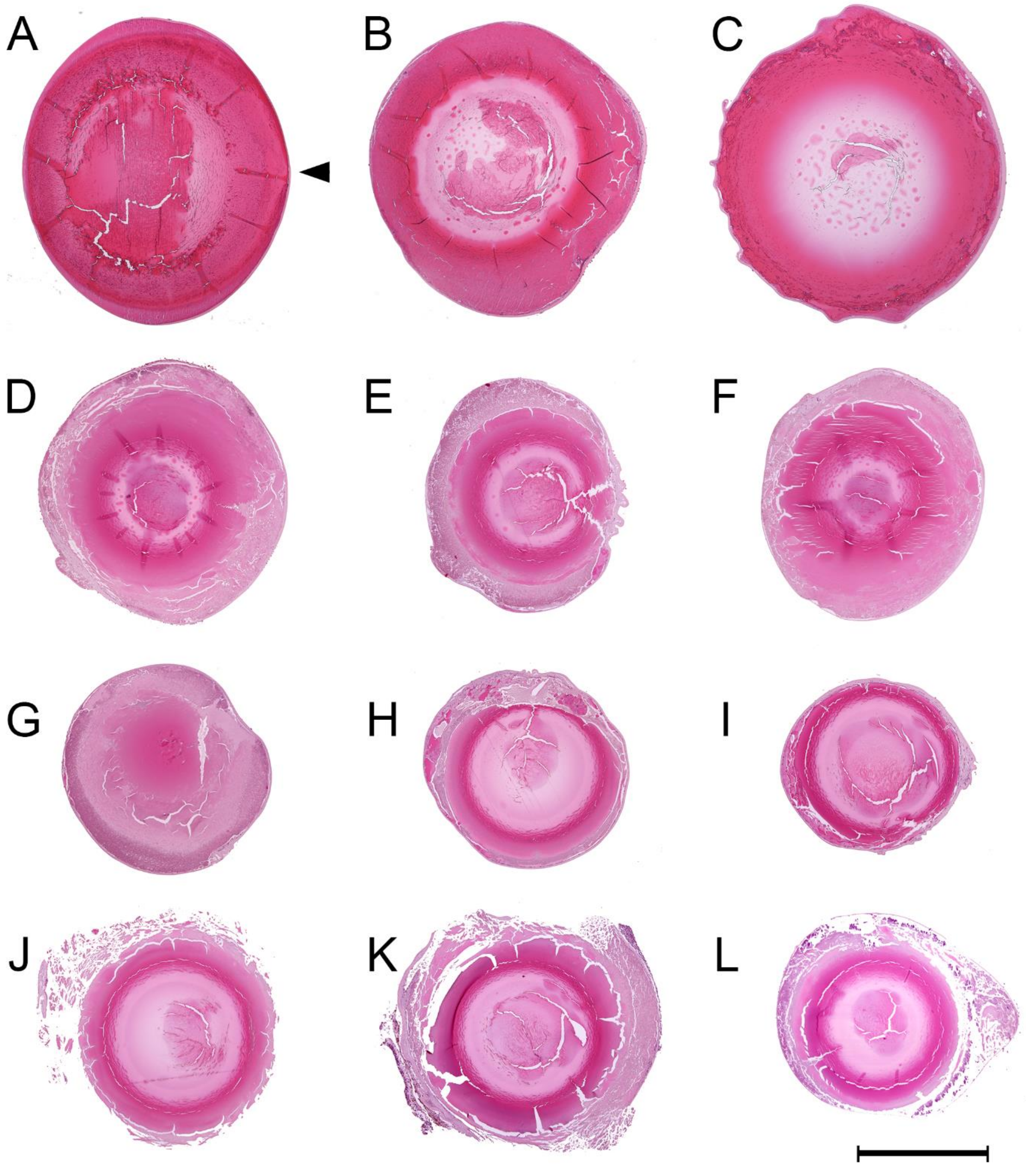

Disease Models & Mechanisms • Supplementary information

Figure S9. Mid-sagittal histological sections of ectopic lenses from 1-1.5 year-old Fbn1-NPCE mice. A control lens **(A)** is provided for comparison. In all cases, the anterior surface of the lens(arrowhead), identified by virtue of its thickened lens capsule, is oriented to the right. **(B-L)** To various degrees, lenses from Fbn1-NPCE mice show gross distortions of shape, loss of eosinophilic material, liquefaction of cortical layers, shrinkage and frank disintegration. Scale bar = 1 mm.

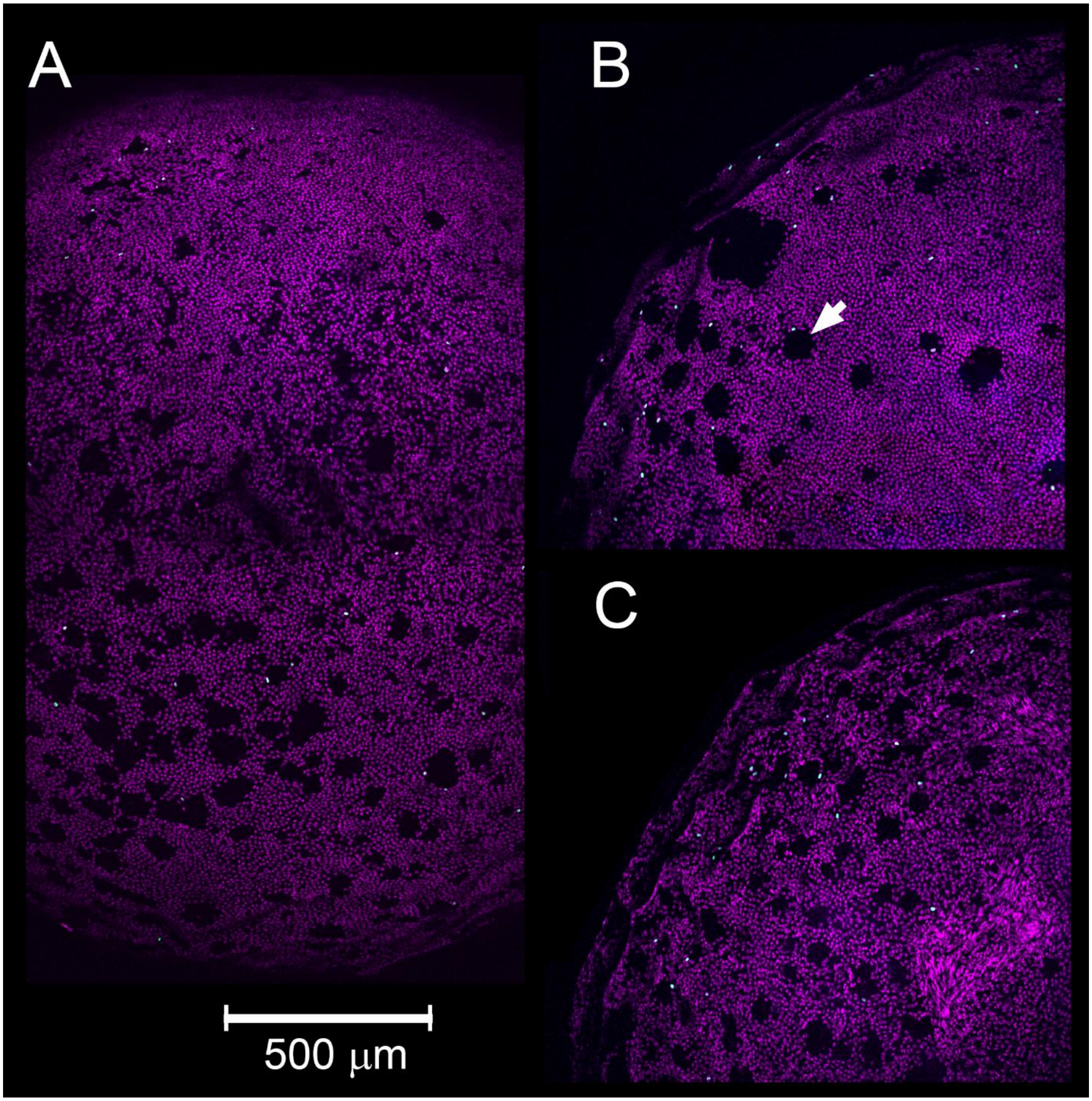

Figure S10. Loss of tissue polarity in ectopic lenses. In control lenses, the epithelial layer covers the anterior surface only. In this ectopic lens from a 14-month-old Fbn1-NPCE mouse, the epithelium has enveloped the entire lens, making the original orientation difficult to discern. Cells cover the “equator” **(A)**, the “anterior” hemisphere **(B)**, and the “posterior” hemisphere **(C)**. Acellular regions (arrow in **B**) are present throughout the epithelium. S-phase cells (green; identified by EdU nuclear labeling) are generally located immediately adjacent to an acellular region. Nuclei (purple) are stained with Draq5. Scale bar = 500 µm.

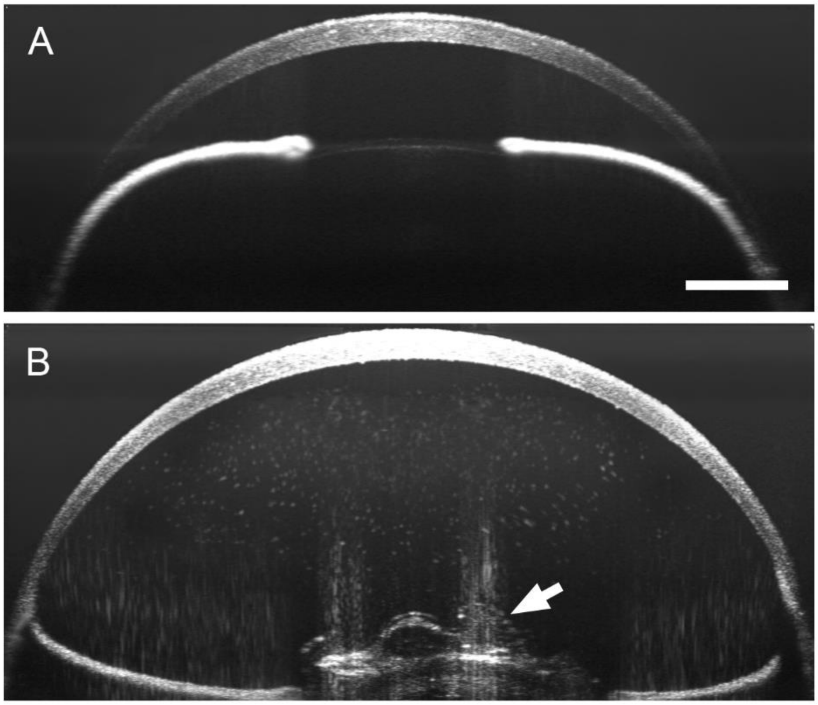

Figure S11. Flocculent material in the anterior chamber of Fbn1-NPCE mouse imaged by OCT. (A) Eye of an 18-month-old control mouse. **(B)** Eye of 21-month-old Fbn1-NPCE mouse. Note the presence of light- scattering material in the anterior chamber and the folded surface of the partially disintegrated ectopic lens protruding through the pupil (arrow). Scale bar = 0.5 mm.

Disease Models & Mechanisms • Supplementary information

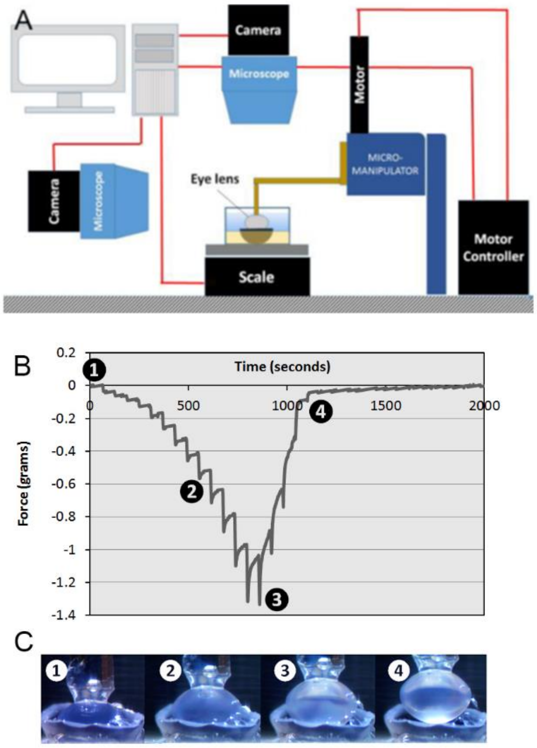

Figure S12. Pull-up procedure for measuring the tensile strength and breaking displacement of zonular fibers. (A) The hemisected eye is immobilized on a balance. A probe is affixed to the posterior surface of the lens and raised in 50 µm increments, while the reduction in weight (at equilibrium, equal to the lifting force) is recorded. **(B)** Representative data from the eye of a 1-month-old wild type mouse. The weight decreases until position 3 (corresponding to a lifting force of approximately 1.1 g (11 mN) and a vertical displacement of about 0.6 mm), at which point the zonular fibers begin to break. By position 4, the fibers have broken, and the weight has returned to baseline. **(C)** Photographs of the lens as it is lifted from the eye (the numbers correspond to those shown in **B**).

Disease Models & Mechanisms • Supplementary information
